# Postoperative Prophylaxis of Cystic Echinococcosis with Albendazole and Praziquantel: A Retrospective Cohort Study

**DOI:** 10.3390/biomedicines14081734

**Published:** 2026-07-31

**Authors:** Galya Popova-Daskalova, Kostadin Kostadinov, Krasimira Eneva, Albena Masarlieva, Dimitar Vuchev, Angel Uchikov, Lyubka Aleksova, Nikolay Nikolov, Tanya Nedkova

**Affiliations:** 1 Department of Infectious Diseases, Parasitology and Tropical Medicine, Section of Parasitology, Medical University of Plovdiv, 4002 Plovdiv, Bulgaria; krasimira.eneva@mu-plovdiv.bg (K.E.); albena.masarlieva@mu-plovdiv.bg (A.M.); dimitar.vuchev@mu-plovdiv.bg (D.V.); 2 “St. George” University Hospital, 4002 Plovdiv, Bulgaria; angel.uchikov@mu-plovdiv.bg (A.U.); lyubka.aleksova@mu-plovdiv.bg (L.A.); nikolay.st.nikolov@mu-plovdiv.bg (N.N.); 3 Department of Social Medicine and Public Health, Medical University of Plovdiv, 4002 Plovdiv, Bulgaria; kostadinr.kostadinov@mu-plovdiv.bg; 4 Environmental Health Division, Research Institute at Medical University of Plovdiv, 15-A “Vasil Aprilov” Blvd., 4002 Plovdiv, Bulgaria; 5 Department of Special Surgery, Medical University of Plovdiv, 4002 Plovdiv, Bulgaria; 6 Faculty of Medicine, Medical University of Plovdiv, 4002 Plovdiv, Bulgaria; tanyanedkova26@gmail.com

**Keywords:** hydatidosis, *Echinococcus granulosus*, combination chemotherapy, anthelmintics, drug tolerability, propensity score, Bayesian analysis, person time incidence

## Abstract

**Background/Objectives**: Cystic echinococcosis is primarily managed surgically, yet postoperative recurrence affects 2–22% of patients despite albendazole prophylaxis. This study describes the tolerability of adding praziquantel to postoperative albendazole and reports recurrence signals where efficacy cannot be inferred. **Methods**: In this single-centre, retrospective, two-arm cohort, 60 patients undergoing hydatid cyst surgery (2024–2026) received albendazole and 30 additionally received praziquantel. The follow-up was substantially shorter in the combination arm (median 10 versus 24 months), and recurrence was first ascertainable only from 12 months, the central limitation. Analyses used standardized mean differences, person time incidence, propensity score and inverse probability weighting, a DAG-based hepatotoxicity model, and a Bayesian Beta-Binomial re-analysis. **Results**: Baseline covariates were markedly imbalanced (14/15 with |SMD| > 0.10). Two recurrences occurred, both in the albendazole arm (3.73/100 person-years) and none in the combination arm’s shorter window. Adverse events were similar (43.3% vs. 50.0%), with no added hepatotoxicity after adjustment (odds ratio 1.45, 95% CI 0.49–4.37). The Bayesian posterior probability of benefit (0.88–0.93) remained below 0.95, a descriptive signal only. **Conclusions**: Albendazole plus praziquantel was well tolerated, but severe baseline imbalance, differential follow-up, and only two events mean efficacy cannot be assessed. The results of our study are hypothesis-generating. Further confirmation requires a prospective, multicentre randomized trial with balanced long-term follow-up.

## 1. Introduction

Cystic echinococcosis (CE), commonly known as hydatidosis, is a significant zoonotic parasitic disease caused by the larval stage of the tapeworm *Echinococcus granulosus*. The life cycle of the parasite involves definitive hosts, primarily domestic dogs and other wild canids, which harbour the adult tapeworms in their small intestines. Intermediate hosts are typically domestic herbivores (sheep, cattle, and goats), in which the larval stage develops as hydatid cysts. Humans act as accidental, aberrant intermediate hosts, typically acquiring the infection through the ingestion of tapeworm eggs via contaminated food, water, or direct contact with definitive hosts. The primary site of anatomical involvement is the liver (60–70%), followed by the lungs (20%), though other organ systems, including the brain, kidneys, and skeletal muscle, can also be affected [1,2]. Structurally, the hydatid cyst wall comprises three distinct layers: the innermost germinal layer, the middle acellular laminated layer, and the host-derived outer adventitial layer (pericyst). When cysts produce viable protocolizes within their fluid, they are termed fertile [3]. The clinical progression of CE is classically slow and asymptomatic, often remaining undetected until the cyst reaches a size sufficient to cause mass effect or severe complications such as rupture or secondary infection [1,3]. The chronic course, potential for multi-organ involvement, and long-term functional impairment after intervention underscore the substantial socioeconomic and public health burden of this infection [1,4].

Cystic echinococcosis is endemic across the Mediterranean basin, Eastern Europe and the Balkans, with Bulgaria among the higher-incidence countries in the European region [4,5]. The World Health Organization-Informal Working Group on Echinococcosis (WHO-IWGE) ultrasound classification (CE1–CE5) provides the imaging-based framework that guides both treatment selection and recurrence monitoring [6,7]. Despite well-defined diagnostic pathways, real-world specialist practice remains heterogeneous, particularly with respect to the use of perioperative chemotherapy and the choice of intraoperative scolicidal agents [8,9]. Emerging nanobiosensor platforms may refine both primary diagnosis and post-treatment surveillance in the future [10].

Although conservative options such as pharmacotherapy (typically albendazole monotherapy) or minimally invasive techniques like Puncture, Aspiration, Injection, Re-Aspiration (PAIR) are utilized, surgical resection remains the principal definitive treatment for active or complicated hydatid cysts [11,12]. However, postoperative recurrence remains a frequent and challenging complication in clinical practice, [13,14]. To mitigate this risk, the World Health Organization (WHO) recommends perioperative chemotherapy, primarily utilizing albendazole, to reduce cyst viability and prevent secondary dissemination [15]. A recent Cochrane systematic review nonetheless emphasizes that the evidence base supporting specific perioperative regimens is methodologically limited, with most controlled studies suffering from small samples and short follow-up [16]. Nonetheless, recurrence remains a concern even among patients receiving standard postoperative prophylaxis [13,17]. While albendazole disrupts microtubule formation in the germinal membrane, its antiparasitic efficacy can be incomplete, allowing some viable protoscolices to survive and subsequently develop into secondary hydatid cysts [18]. In contrast, praziquantel exhibits rapid and potent scolicidal activity in vitro and is highly effective against the adult tapeworms in definitive hosts. Its mechanism of action involves increased calcium influx across the parasite tegument, resulting in severe tegumental damage and spastic paralysis of protoscolices [19]. Accumulating clinical and experimental evidence suggests that albendazole–praziquantel combination therapy provides more rapid and effective disease control than albendazole alone, highlighting its potential value in postoperative management [19,20,21,22,23]. A formal systematic review and meta-analysis of medical treatment for CE also suggested superior efficacy of combination regimens compared with benzimidazole monotherapy, although the pooled analysis was limited by substantial between-study heterogeneity [24].

Despite this mechanistic and experimental rationale, the clinical evidence base for adding praziquantel to albendazole after surgery remains thin. The available studies are few, heterogeneous in dose and duration, largely preoperative or parasitological in focus, and rarely address postoperative recurrence with explicit attention to the confounding and follow-up asymmetries that observational cohorts entail [16,19,24]. Experimental and in vitro work continues to document a scolicidal advantage for the combination, including recent nanoformulation studies [25,26], yet it is unknown whether this pharmacological synergy translates into a measurable postoperative benefit in patients at a tolerable safety cost, and real-world practice remains correspondingly heterogeneous [8,9]. Against this gap, the present study describes postoperative outcomes, tolerability, and recurrence signals among patients receiving albendazole alone or albendazole plus praziquantel after surgical resection for cystic echinococcosis. Because allocation was non-randomized and follow-up was systematically shorter in the combination arm and because recurrence in cystic echinococcosis can arise years after surgery, the study was framed a priori as a hypothesis-generating safety, tolerability, and feasibility analysis rather than as a test of comparative efficacy. Therefore, the recurrence contrast is reported as a directional signal whose interpretation is bounded by the design.

## 2. Materials and Methods

### 2.1. Study Design and Setting

This was a single-centre, retrospective, observational two-arm cohort study comparing postoperative albendazole monotherapy with combined albendazole plus praziquantel prophylaxis. The study was based on the medical records of patients treated and followed for cystic echinococcosis at “St. George” University Hospital, Plovdiv, Bulgaria, between April 2024 and April 2026. Patients who underwent surgery at another hospital but were referred to our hospital for follow-up were also included in the study. Allocation to the two prophylactic regimens was determined by treating clinicians based on clinical judgement (cyst fertility, suspected or ascertained hydatid fluid spillage, recurrent cysts) and contraindications rather than by randomization. The comparison is therefore observational, and the analytic strategy was directed throughout at the confounding, and ascertainment biases this design entails. Throughout the enrolment window, patients were managed by the same hepatobiliary and thoracic surgical teams under a stable institutional protocol for perioperative chemoprophylaxis, intraoperative scolicidal application, residual cavity management, and follow-up scheduling, so that operative technique and postoperative management were consistent across the study period. The study is reported in accordance with the Strengthening the Reporting of Observational Studies in Epidemiology (STROBE) statement for cohort studies [27].

### 2.2. Participants and Eligibility Criteria

The source population comprised all consecutive adults surgically treated for cystic echinococcosis who received postoperative chemoprophylaxis and were followed up at the study centre during the enrolment window. Eligibility criteria were applied at the level of the medical record. Inclusion criteria were age ≥ 19 years, surgical treatment for hepatic, pulmonary, and/or abdominal cystic echinococcosis, and complete surgical removal of all hydatid cysts. Exclusion criteria were age < 19 years, surgical treatment of cystic echinococcosis at other anatomical locations, incomplete cyst removal in cases of multiple cystic echinococcosis, impaired hepatic or renal function, hematological disorders, and pregnancy. Patients with epilepsy or cardiac rhythm and conduction disorders were not allocated to the praziquantel-containing regimen, reflecting the established contraindications to praziquantel. This constituted a deterministic, indication-driven component of allocation and is acknowledged as a source of confounding by indication.

### 2.3. Prophylactic Regimens

All patients received albendazole 400 mg twice daily, taken after a meal, for 2 to 4 months after surgical treatment. Thirty patients additionally received praziquantel 25 mg/kg/day, taken after a meal, for 10 days, concomitantly with albendazole at the beginning of prophylaxis and within three months after surgical treatment. The praziquantel dose (25 mg/kg/day) was lower than the most recent WHO recommendation, as the management protocol on which these records are based predated that guidance and followed the historical regimen of Cobo et al. [28].

### 2.4. Outcomes, Variables and Data Sources

The primary outcome was postoperative disease recurrence, operationally defined as the imaging-detected appearance of a new cyst within the surgical bed or residual cavity, supported by a documented rise in anti-*Echinococcus* IgG titre over serial measurements rather than by a single positive serological result. Recurrence status was recorded at the scheduled 12-, 18-, and 24-month visits. The endpoint was therefore interval-censored and was directly ascertainable only from 12 months onward. Secondary outcomes were the safety and tolerability of the regimens (transaminase elevation, anemia, gastrointestinal events, and new-onset eosinophilia), the time to obliterate the postoperative residual cavity, and the longitudinal anti-*Echinococcus* IgG trajectory. Hepatotoxicity was graded using the peak on-treatment fold elevation of aspartate aminotransferase (AST) and alanine aminotransferase (ALT) relative to an upper limit of normal (ULN) of 40 U/L for both enzymes (≤2 × ULN; >2–3 × ULN; >3 × ULN). New-onset eosinophilia was defined as a baseline eosinophil fraction ≤5% rising to >5% at any on-treatment visit. All variables were abstracted from the hospital’s clinical, laboratory, and imaging records. Anti-*Echinococcus* IgG was measured using enzyme-linked immunosorbent assay (ELISA); hematology and liver enzymes were measured at the hospital laboratory; cyst characteristics and cavity status were assessed through abdominal ultrasound, chest radiography, and computed tomography. Because all data were drawn from a single institution’s standard assessment pathway, the measurement methods were comparable across the two arms. Per-organ cyst counts (hepatic, pulmonary, abdominal) and per-cyst size band counts (≤100 mm versus >100 mm) were abstracted from the operative and discharge documentation; the cyst-level partition therefore reflects every resected cyst rather than only the largest per patient.

### 2.5. Follow-Up and Outcome Ascertainment

Complete blood count, differential white cell count, and liver enzymes were assessed every 15 days or monthly during albendazole intake, and a structured history of adverse effects attributable to albendazole or praziquantel was recorded at each visit. Patients were monitored for recurrence using serial ELISA for anti-*Echinococcus* IgG and imaging (abdominal ultrasound, chest radiography, and computed tomography) every 3–6 months. Imaging was performed and interpreted as part of routine clinical care by the hospital’s radiologists, who were not formally blinded to treatment allocation. Because the study is a retrospective abstraction of these records, no independent blinded re-reading was undertaken. This is acknowledged as a potential source of information bias and is mitigated by the objective, size-based definition of cavity obliteration and by the requirement that a recurrence be corroborated by a documented rise in serological titre rather than by imaging alone. Follow-up duration differed systematically between the arms by virtue of the management protocol: the combination-treated patients were managed over a shorter window and were therefore followed for a median of 10 months (range 6–18) compared with 24 months (range 6–24) in the monotherapy arm. This differential, non-random follow-up is the central design limitation and underlies the structural, protocol-driven missingness in the later visit blocks. Missingness of this kind was not treated as random and was not imputed. Patients whose follow-up ended before the first recurrence assessment at 12 months were treated as right-censored at their last documented assessment, with no event observed rather than confirmed recurrence-free.

### 2.6. Bias and Study Size

Several sources of bias follow directly from the design and were addressed analytically rather than by exclusion. Non-randomized, indication-driven allocation introduces confounding by indication, addressed through standardized mean differences, propensity score and inverse probability weighting (for adequately powered safety endpoints), and explicit reporting of residual confounding under limited covariate overlap. The retrospective abstraction of routinely collected data introduces the potential for selection and information bias, mitigated through the application of uniform eligibility criteria to consecutive records and through the single-institution, protocol-standardized assessment pathway. Differential follow-up introduces ascertainment bias for time-dependent endpoints, addressed by per-visit denominator tabulation, person time incidence rates, a primary window analysis restricted to the subcohort that reached the 12-month recurrence ascertainment landmark, fixed horizon landmark analyses, and tipping point sensitivity analysis. The accrual of only two recurrence events, both confined to the albendazole arm, produces a complete separation pattern that renders frequentist hypothesis tests and time-to-event models uninformative. This was anticipated in the analysis plan and motivated the a priori decision to summarize the recurrence endpoint within a Bayesian conjugate Beta-Binomial framework, in which finite posterior distributions and a directly interpretable probability of benefit remain available under zero-event cells. No formal a priori sample size calculation was performed. Our study comprised all consecutive eligible patients treated during the two-year window, yielding a convenience cohort of 60 patients (30 per arm) that is acknowledged to be underpowered for the recurrence endpoint.

### 2.7. Statistical Analysis

Statistical analysis was performed using R software (version 4.5.1; R Foundation for Statistical Computing, Vienna, Austria). All statistical tests were two-tailed, and a *p*-value < 0.05 was considered statistically significant. Given the non-randomized design, between-arm *p*-values are reported as descriptive summaries of the observed distributions rather than as tests of a sampling null, and effect estimates with confidence or credible intervals are emphasized over significance thresholds throughout. To improve readability, the present section summarizes the analytic strategy at the level of principle. The full technical specification of the propensity score, inverse probability weighting, directed acyclic graph, landmark, tipping point, and Bayesian procedures, together with the priors, estimands, and diagnostic outputs, is provided in Supplementary File S1 (Section S9), and the complete annotated code is provided as Supplementary File S2.

Baseline characteristics of the two treatment cohorts were summarized as mean ± standard deviation (SD) for normally distributed continuous variables, median and interquartile range (IQR) for non-normally distributed continuous variables, and frequencies with percentages for categorical variables. The choice of test was governed by the data: continuous variables were compared using Student’s (or Welch’s) *t*-test when both arms satisfied the Shapiro–Wilk normality criterion and otherwise using the Mann–Whitney U test. Categorical variables were compared using Pearson’s chi-square test, with Fisher’s exact test substituted whenever more than 20% of expected cell counts fell below five or any expected count fell below one. Each continuous comparison was accompanied by an effect size consistent with the selected test (mean difference with 95% CI for the *t*-test path; Hodges–Lehmann location shift with 95% CI for the rank-based path). Because treatment allocation was non-randomized, baseline covariate balance was assessed using standardized mean differences (SMDs), with a threshold of |SMD| > 0.10 indicating potential imbalance. The SMD, rather than the significance test, was treated as the design-appropriate balance metric (Supplementary File S1, Table S1 and Figure S1).

Recurrence-free survival was analyzed using Kaplan–Meier curves and compared across groups using the log-rank test. Owing to differential follow-up duration and the small number of events (n = 2), per-visit attendance denominators were tabulated and visualized, and person time incidence rates per 100 person-years with exact Poisson 95% CIs were calculated. The recurrence probability at 12, 18, and 24 months was derived as 1 − KM (Supplementary File S1, Table S3). As the primary efficacy comparison anchored on the data overlap window, the recurrence contrast was additionally reported within the subcohort that reached the 12-month landmark (the earliest visit at which the endpoint is directly ascertainable), with the absolute risk difference summarized on the Newcombe hybrid score 95% CI. Absolute between-arm effects across endpoints were consolidated as risk differences (Newcombe 95% CI) and risk ratios (Katz logarithmic 95% CI, with a 0.5 continuity correction where a cell contained no events) into a single endpoint table (Supplementary File S1, Table S2). To assess robustness under unequal follow-up, a tipping point sensitivity analysis was performed over the unascertained patients (those with <12 months of follow-up), simulating a worst-case scenario in which all such patients had recurred. A semi-parametric interval-censored proportional hazards model was fitted as a sensitivity analysis. With zero events in the combination arm this model separated and was therefore treated as confirmation that frequentist time-to-event modelling of this endpoint was uninformative.

To address the complete absence of events in the combination arm, which renders the Fisher exact test uninformative and causes the frequentist time-to-event models to diverge, the binary endpoints (recurrence, any adverse event, and elevated transaminases) were additionally re-analyzed within a Bayesian conjugate Beta-Binomial framework. Each arm’s event probability was assigned an independent Beta prior and updated to a closed-form Beta posterior. The posterior distributions of the risk difference, risk ratio, and odds ratio (combination versus albendazole) were obtained through Monte Carlo sampling (200,000 draws). A uniform Beta (1, 1) prior served as the primary analysis and a Jeffreys Beta (0.5, 0.5) prior as a prior sensitivity check. The posterior probability of benefit—the probability that the combination carried the lower event probability—was reported as a directly interpretable summary that, unlike the separated frequentist models, remains finite under a zero-event arm and was interpreted against conventional Bayesian evidence thresholds (a posterior probability of 0.95 corresponding to moderate evidence and 0.975 to strong evidence).

For safety outcomes, propensity score (PS) adjustment and inverse probability of treatment weighting (IPW) were applied to control for baseline imbalances, restricted to the adequately powered adverse event endpoints rather than the two-event recurrence endpoint. A parsimonious logistic regression model estimated propensity scores from age, number of cysts, baseline cyst size, rural residence, and baseline AST. Covariate overlap was diagnosed by plotting propensity score densities to establish the region of common support (Supplementary File S1, Figure S3); post-weighting balance was verified using weighted SMDs; and the inverse-probability-weighted effective sample size (Supplementary File S1, Table S5 and Figure S4) and adjusted odds ratios (ORs) with 95% CIs were computed. Within-arm transaminase elevations from baseline to peak on-treatment values were assessed using paired Wilcoxon signed-rank tests. Longitudinal transaminase burden over the active monitoring window (0–3 months) was quantified using the incremental Area Under the Curve (AUC) above baseline (trapezoidal rule), compared across arms using Mann–Whitney U tests and baseline-adjusted linear regression. A linear mixed effects model (LMM) with a random patient intercept was fitted to AST values over the active treatment period (0.5–3 months) to evaluate the group-by-time interaction, adjusted for baseline levels. The adjustment set for the hepatotoxicity contrast was specified as a priori through a directed acyclic graph (Supplementary File S1, Figure S6). Under the encoded causal assumptions, the minimal sufficient adjustment set for the total effect of treatment on hepatotoxicity comprises baseline AST and age, which together define the prespecified primary adjusted model (treatment + baseline AST + age). The earlier {baseline AST, baseline ALT} specification was retained as a sensitivity model, and a kitchen sink {baseline AST, baseline ALT, age} specification was added for completeness. To gauge robustness to unmeasured confounding, the E-value of the DAG-aligned adjusted odds ratio was computed for both the point estimate and the confidence interval bound closer to the null, following the closed-form expression of VanderWeele and Ding [29].

Time to obliterate the postoperative residual cavity was estimated using Kaplan–Meier methods, with patients whose cavity had not obliterated censored at their last documented follow-up. Because the all-time log-rank comparison is confounded by the arms’ differential follow-up, a follow-up-robust landmark analysis was performed at fixed horizons (6 and 12 months), comparing the estimated obliteration probabilities using a two-sample z-test with Greenwood standard errors. Interpretation was anchored on the 6-month horizon, at which both arms were fully observed.

Missing values arising from the differential, protocol-driven follow-up were regarded as structural (missing not at random by construction) rather than as random attrition and were not imputed. Analyses were confined to the visits each arm reached. The structural-versus-sporadic missingness pattern was characterized explicitly to forestall its misreading as random loss to follow-up (Supplementary File S1, Section S7 and Figure S5). The complete annotated R script that produced every table and figure is provided as Supplementary File S2.

## 3. Results

### 3.1. Patient Demographics and Baseline Covariate Balance

**Figure 1:**
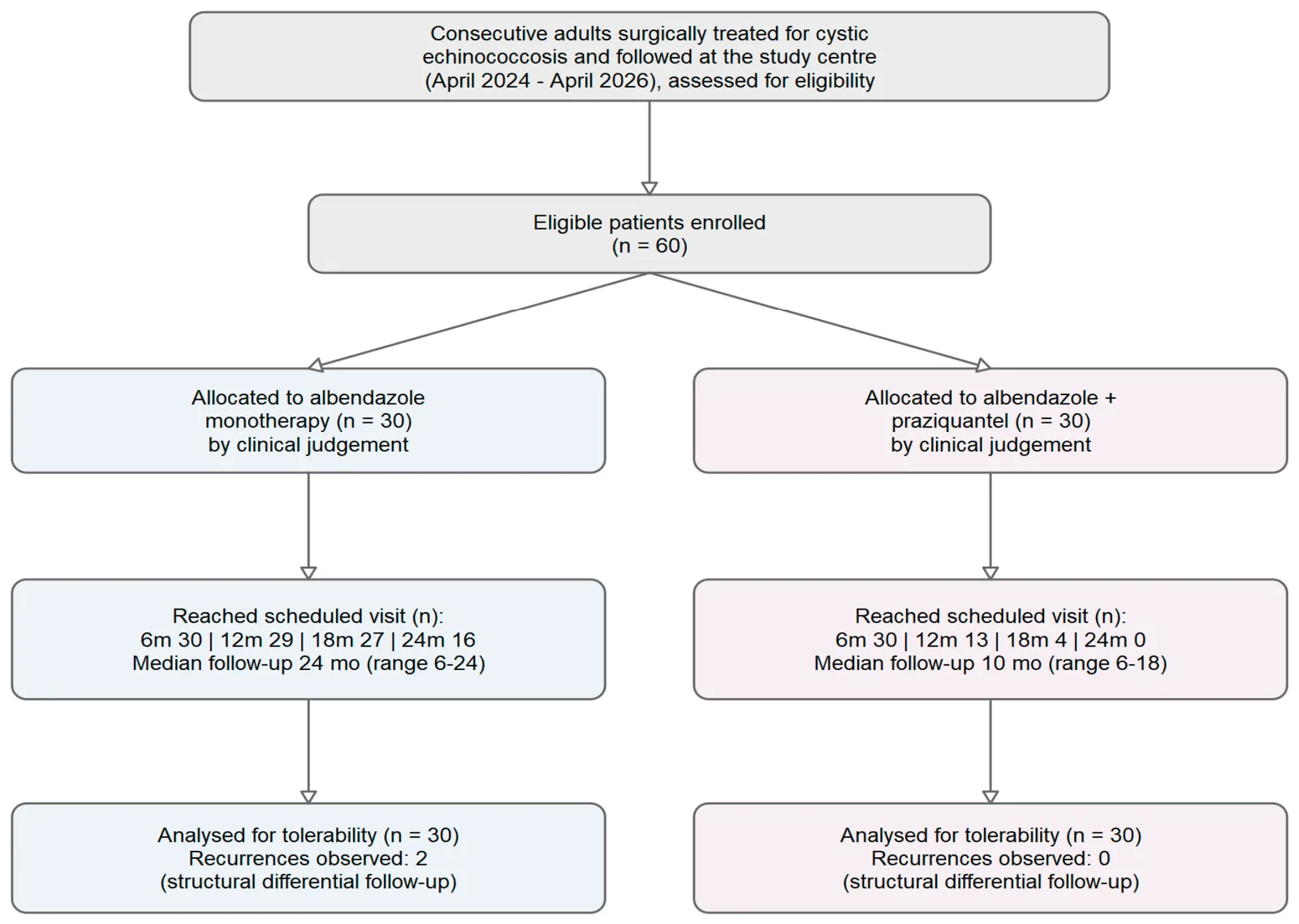
STROBE participant flow diagram. Consecutive eligible adults were allocated by clinical judgement to albendazole monotherapy or albendazole plus praziquantel, with the number reaching each scheduled follow-up visit (6, 12, 18, and 24 months) shown per arm. The progressive fall in the combination arm’s denominators (13 at 12 months, 4 at 18 months, and 0 at 24 months) reflects the structural, protocol-driven differential follow-up rather than random loss to follow-up. Both recurrences occurred in the albendazole arm.

A total of 60 patients were enrolled, with 30 receiving postoperative albendazole monotherapy and 30 receiving the combination of albendazole and praziquantel. The participant flow from eligibility through allocation, follow-up reached at each scheduled visit, and analysis is summarized in Figure 1. The mean age of the entire cohort was 43.9 ± 15.1 years (range 19–77). Demographics and clinical characteristics of the two groups are summarized in Table 1.

Because the allocation of patients to treatment arms was non-randomized, baseline comparability was formally assessed using standardized mean differences. The full balance table covering all 15 measured baseline covariates is reported in Supplementary File S1 (Table S1) and visualized as a Love plot in Supplementary File S1 (Figure S1). Fourteen of the 15 covariates exceeded the conventional balance threshold of |SMD| > 0.10, indicating that the two arms were not exchangeable at baseline. The single balanced covariate was the per-patient hepatic cyst count (SMD = −0.03). The combination group showed a markedly higher baseline transaminase burden (baseline AST 31.7 ± 18.5 vs. 24.6 ± 9.7 U/L, SMD = −0.478; baseline ALT 37.6 ± 26.9 vs. 28.3 ± 25.2 U/L, SMD = −0.357, *p* = 0.018), larger baseline cyst size, a higher proportion of fertile cysts (70.0% vs. 53.3%), and a substantially greater abdominal cyst burden (mean 0.7 ± 2.8 vs. 0.1 ± 0.3 abdominal cysts per patient, SMD = −0.335); this last imbalance was driven by two patients in the combination arm with disseminated intra-abdominal disease and is examined further in the cyst-level analysis below.

### 3.2. Cyst Burden and Characteristics

A total of 118 hydatid cysts were surgically removed from the 60 patients: 47 cysts in the albendazole group and 71 cysts in the combination group. Cyst characteristics are summarized at three complementary levels in Table 2. The per-patient block reproduces the conventional descriptors (cysts per patient and the diameter of the largest cyst per patient). The cyst-level organ block partitions all 118 resected cysts by anatomical site and the cyst-level size block partitions them by maximum diameter (≤100 mm versus >100 mm), abstracted from the operative and discharge documentation rather than from the largest cyst proxy alone.

The cyst-level partition shows that the combination arm carried a notably higher absolute and relative abdominal cyst burden (22 of 71 cysts, 31.0%) than the albendazole arm (2 of 47 cysts, 4.3%). This imbalance is concentrated in two patients with disseminated intra-abdominal disease (10 and 12 abdominal cysts, respectively) and propagates upward into the per-patient SMD reported in Supplementary File S1, Table S1. At the cyst level, the distribution of cyst sizes did not differ between arms (Fisher’s exact *p* = 0.547; chi-square *p* = 0.453), and the per-patient largest cyst dichotomy similarly showed no between-arm difference (*p* = 0.592). Nineteen patients (63.3%) in the albendazole group and sixteen (53.3%) in the combination group underwent surgery for a single hydatid cyst.

### 3.3. Operative Techniques and Scolicidal Management

Among the 46 patients who had a hepatic component involved (24 in the albendazole group and 22 in the combination group), partial pericystectomy was the most frequently performed procedure (Table 3). No statistically significant difference in operative technique distribution was observed between the two groups (Fisher’s exact *p* = 0.722). Laparoscopy was utilized for one patient in each group, while the rest underwent open laparotomy.

For pulmonary hydatidosis, patients were treated using atypical lung resection. Scolicidal agents used intraoperatively included iodine, hypertonic sodium chloride, iodasept, braunol, and hibitane. Residual cavity management was addressed via capitonage, omentoplasty, and drainage.

#### 3.3.1. Hydatid Fluid and Protoscolex Viability

Microscopic examination of hydatid fluid was performed for 21 surgically removed cysts to evaluate the presence of protoscolices. Of these, five of seven examined samples (71.4%) in the albendazole group and nine of 14 examined samples (64.3%) in the combination group were positive for protoscolices (Table 4).

In the full cohort, using the presence of a fertile cyst at baseline as a proxy for parasite viability (defined as a cyst whose hydatid fluid contained protoscolices on microscopic examination or whose germinal layer carried morphologically intact protoscolices on histological examination), 16 patients (53.3%) in the albendazole arm and 21 patients (70.0%) in the combination arm had fertile cysts (Pearson’s chi-square *p* = 0.184). Because direct microscopy was performed on only a subset of resected cysts and the dataset does not carry a per-cyst microscopy indicator, the fertility variable is a patient-level proxy derived from the available microscopic and histological notes rather than a complete cyst-level assay, which is acknowledged as a limitation.

#### 3.3.2. Postoperative Cavity Obliteration and Longitudinal Serology

**Figure 2:**
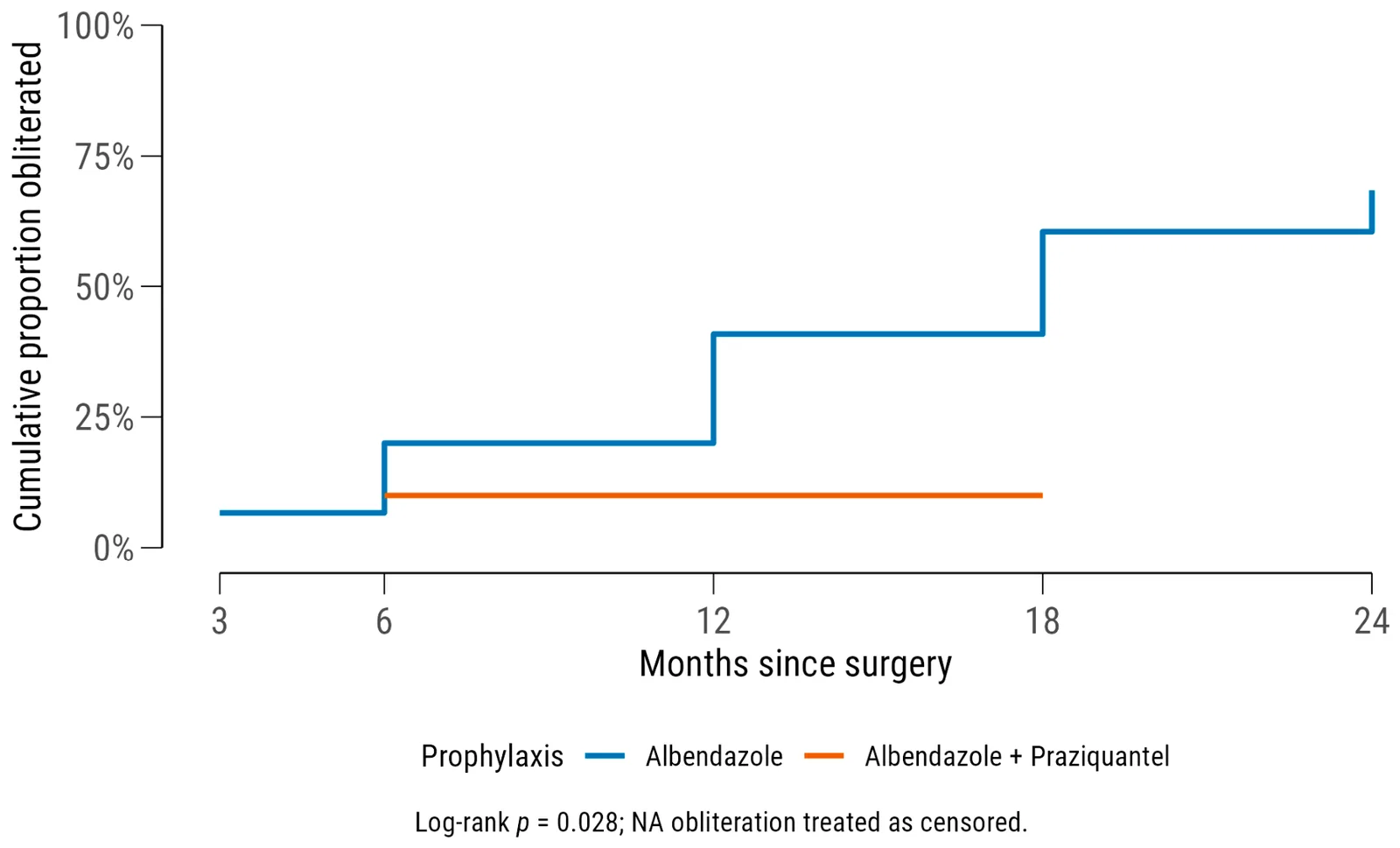
Cumulative proportion of patients achieving obliteration of the postoperative residual cavity over time, by prophylaxis arm (albendazole alone versus albendazole plus praziquantel), estimated using the Kaplan–Meier method with patients whose cavity had not obliterated censored at their last documented imaging follow-up. Because the all-time comparison is confounded by the arms’ differential follow-up, interpretation is anchored on the 6-month landmark at which both arms remained fully observed (see text). The *x*-axis shows months since surgery and the *y*-axis the cumulative obliteration probability.

Because the all-time log-rank comparison of time to cavity obliteration is confounded by the arms’ differential follow-up, interpretation was anchored a priori on the 6-month landmark at which both arms remained fully observed. At the 6-month landmark, the estimated cavity obliteration rate was 20.0% in the albendazole arm and 10.0% in the combination arm, which did not differ statistically (difference 10.0 percentage points, z = 1.10, *p* = 0.271): the design-appropriate, follow-up-robust contrast therefore shows no demonstrated between-arm difference. For descriptive completeness, the crude comparison over the full observation window yielded 18 of 30 patients (60.0%) with documented obliteration in the albendazole arm (median time 18 months, 95% CI 12 to not estimable) versus three of 30 (10.0%) in the combination arm (crude log-rank chi-square = 4.839, *p* = 0.028), and the 12-month landmark contrast was 40.9% versus 10.0% (difference 30.9 pp, z = 2.91, *p* = 0.004). Both of these latter contrasts are driven by the combination arm’s truncation to a sparse, selected long-followed remnant (median follow-up 10 months, with most patients censored at 6–10 months) and are presented for transparency only and they should not be interpreted as evidence of a between-arm difference in obliteration biology. Cumulative obliteration curves over the full observation window are shown in Figure 2.

**Figure 3:**
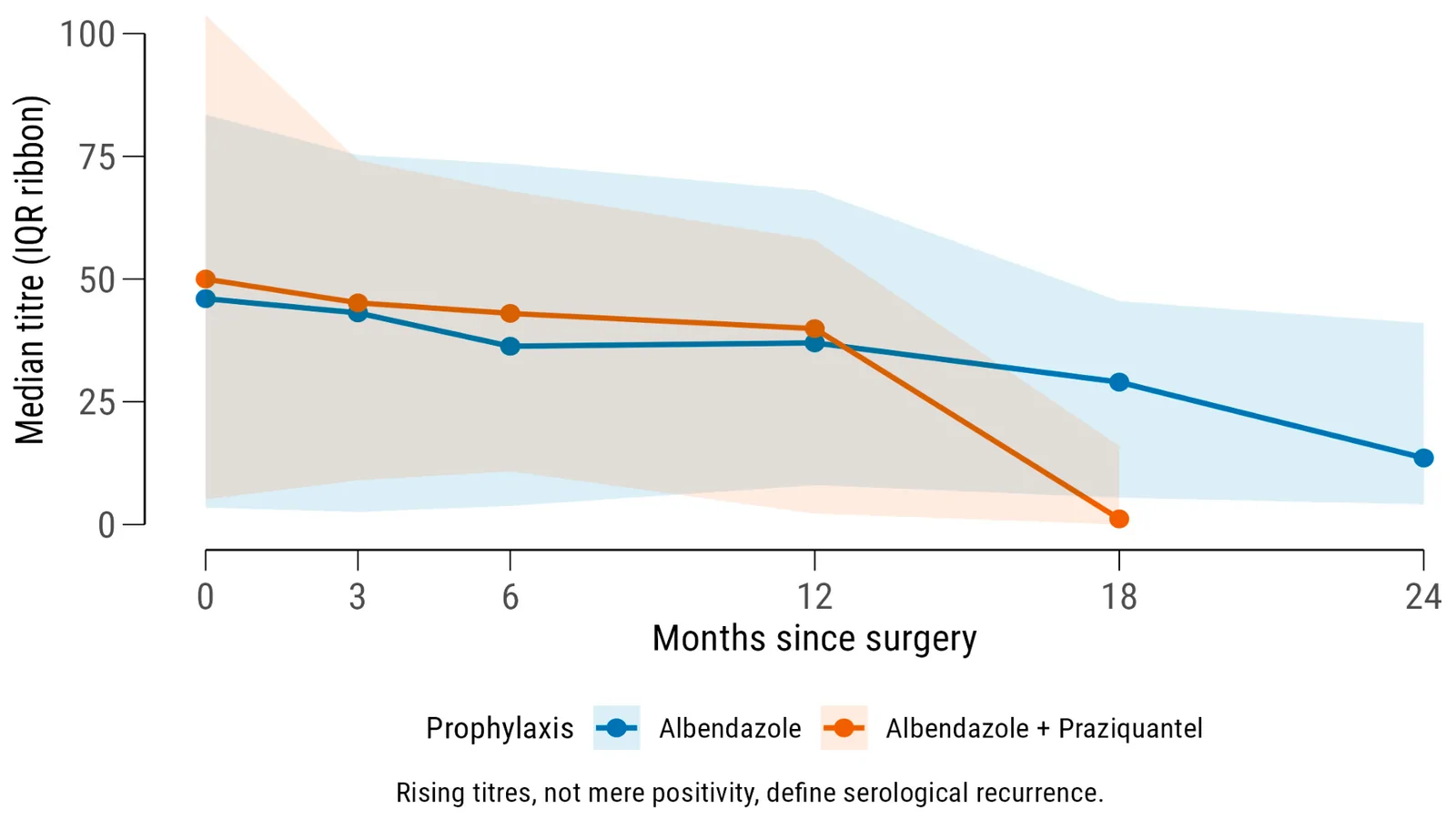
Longitudinal anti-*Echinococcus* immunoglobulin G antibody titre trajectories by prophylaxis arm (albendazole alone versus albendazole plus praziquantel). Solid lines show the median titre, and shaded ribbons show the interquartile range (IQR) at each scheduled visit from baseline to 24 months. The y-axis shows the enzyme-linked immunosorbent assay titre/index, and the x-axis shows months since surgery. Denominators decline over time because of the differential, protocol-driven follow-up; consequently, later time points in the combination arm are based on few patients.

Longitudinal serological monitoring of anti-*Echinococcus* IgG titres showed a gradual decline in both arms. In the albendazole group, median titres decreased from 46.0 (IQR 22.8–104.7) at baseline to 43.1 at 3 months, 36.3 at 6 months, 37.0 at 12 months, 29.0 at 18 months, and 13.6 at 24 months. In the combination group, median titres decreased from 50.0 (IQR 18.0–156.5) at baseline to 45.2 at 3 months, 43.0 at 6 months, 39.9 at 12 months and 1.13 at 18 months. The baseline-to-last-visit change in titres did not differ significantly between the arms (median change: −9.1 [IQR −51.4 to −0.4] in the albendazole group vs. −14.7 [IQR −52.7 to −1.8] in the combination group; Mann–Whitney U *p* = 0.425). At the last available follow-up, serology status was positive in 23 (76.7%) and negative in five (16.7%) albendazole patients (with two in the grey zone), compared to 27 positive (90.0%) and three negative (10.0%) patients in the combination group (Fisher’s exact *p* = 0.365). In both patients with confirmed recurrence the titre trajectory subsequently reversed direction, providing serological corroboration of the imaging-detected event (see Section on disease recurrence below). The full median titre trajectories with interquartile range ribbons are shown in Figure 3.

### 3.4. Safety and Tolerability

Adverse events were reported in 13 patients (43.3%) in the albendazole group and 15 patients (50.0%) in the combination group, showing no statistically significant difference in crude rates (Pearson’s chi-square *p* = 0.605; Table 5). Propensity-score-adjusted logistic regression yielded an adjusted odds ratio (OR) of 1.09 (95% CI 0.33–3.57, *p* = 0.890) for any adverse event. Sensitivity analysis using inverse probability of treatment weighting (IPW) similarly showed no significant difference (OR 0.95, 95% CI 0.46–1.96, *p* = 0.882). Absolute risk differences for the consolidated endpoint set, including 95% Newcombe and Katz intervals, are reported in Supplementary File S1, Table S2.

Both groups experienced highly significant increases in transaminases from baseline to peak on-treatment levels (paired Wilcoxon signed-rank tests: AST increased from median 22 to 35.5 U/L in albendazole, *p* < 0.001, and from 25.5 to 38 U/L in combination, *p* = 0.005; ALT increased from median 17 to 40 U/L in albendazole, *p* < 0.001, and from 33 to 40 U/L in combination, *p* = 0.005).

To determine whether the addition of praziquantel increased hepatotoxicity beyond what baseline transaminase differences would predict, the DAG-implied primary adjusted model (treatment + baseline AST + age; Supplementary File S1, Figure S6) yielded an odds ratio of 1.45 (95% CI 0.49–4.37, *p* = 0.502) for elevated transaminases. The corresponding sensitivity specifications agreed directionally: the prior {treatment + baseline AST + baseline ALT} model gave OR 1.40 (95% CI 0.47–4.22, *p* = 0.539), the {treatment + baseline AST + baseline ALT + age} model gave OR 1.48 (95% CI 0.49–4.57, *p* = 0.486), and the PS-adjusted model gave OR 1.60 (95% CI 0.48–5.59, *p* = 0.449). To gauge sensitivity to unmeasured confounding, the E-value of the DAG-aligned point estimate was 2.26, indicating that an unmeasured confounder would need to be associated with both the combination regimen and elevated transaminases by an odds ratio of at least 2.26, above and beyond the measured covariates, to move the point estimate to the null. The 95% confidence interval for this odds ratio (0.49–4.37), however, already includes the null. No unmeasured confounding is therefore required for the data to be compatible with no added hepatotoxicity, and an E-value for the confidence limit is consequently not interpretable as evidence of robustness and is not reported. The point estimate E-value is presented only as a descriptive gauge of the point estimate’s fragility, and the safety interpretation rests on the confidence interval spanning the null rather than on any claim of robustness. Propensity score overlaps and the region of common support are visualized in Supplementary File S1, Figure S3.

**Figure 4:**
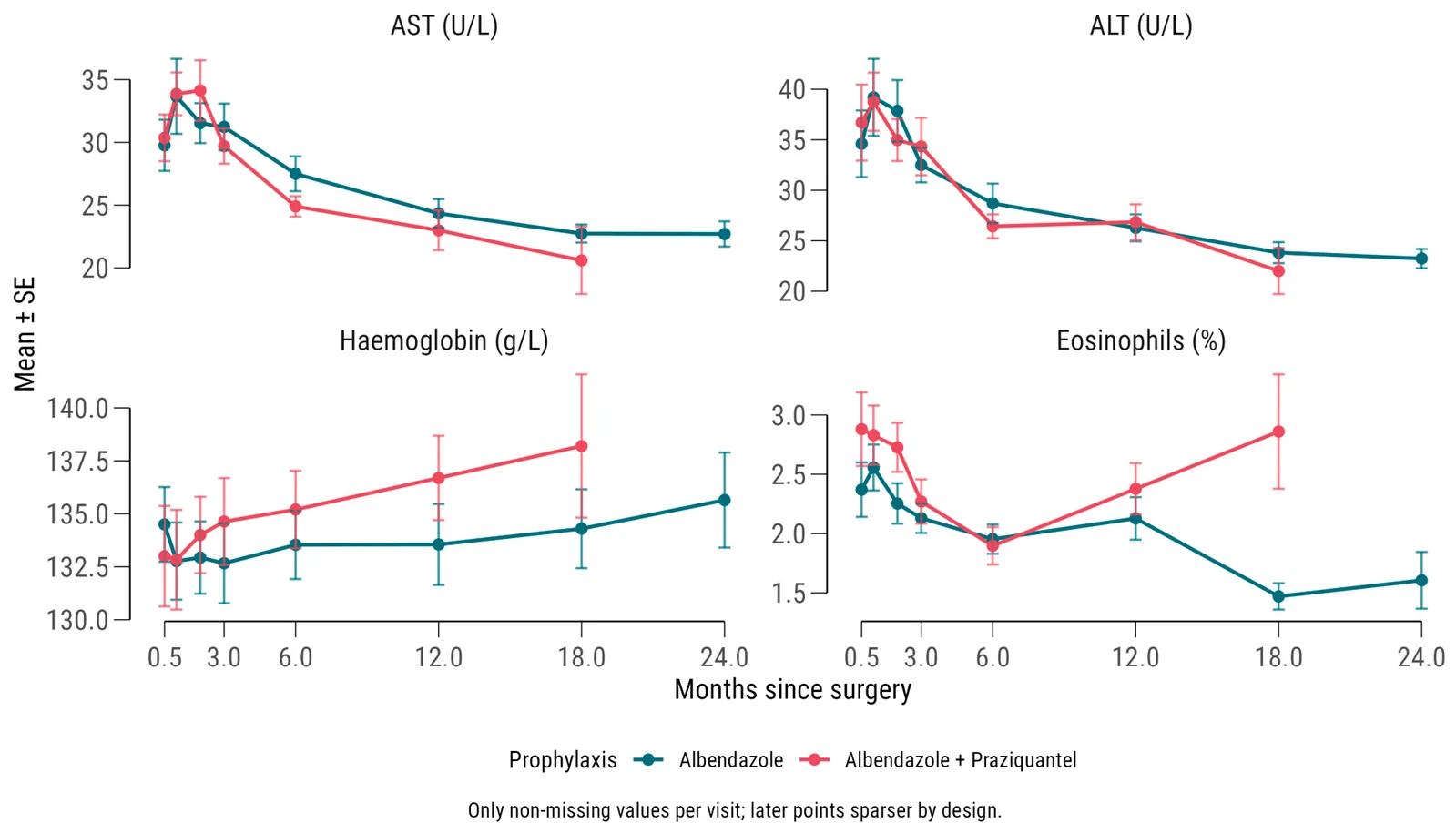
Longitudinal laboratory trajectories over the active treatment period by prophylaxis arm (albendazole alone versus albendazole plus praziquantel), shown as mean ± standard error (SE) at each scheduled visit for four analytes: aspartate aminotransferase (AST), alanine aminotransferase (ALT), hemoglobin, and eosinophils. Each panel plots the analyte value (*y*-axis) against months since surgery (*x*-axis). AST and ALT are expressed in U/L (upper limit of normal 40 U/L), hemoglobin in g/L, and eosinophils as a percentage of the differential white cell count.

**Figure 5:**
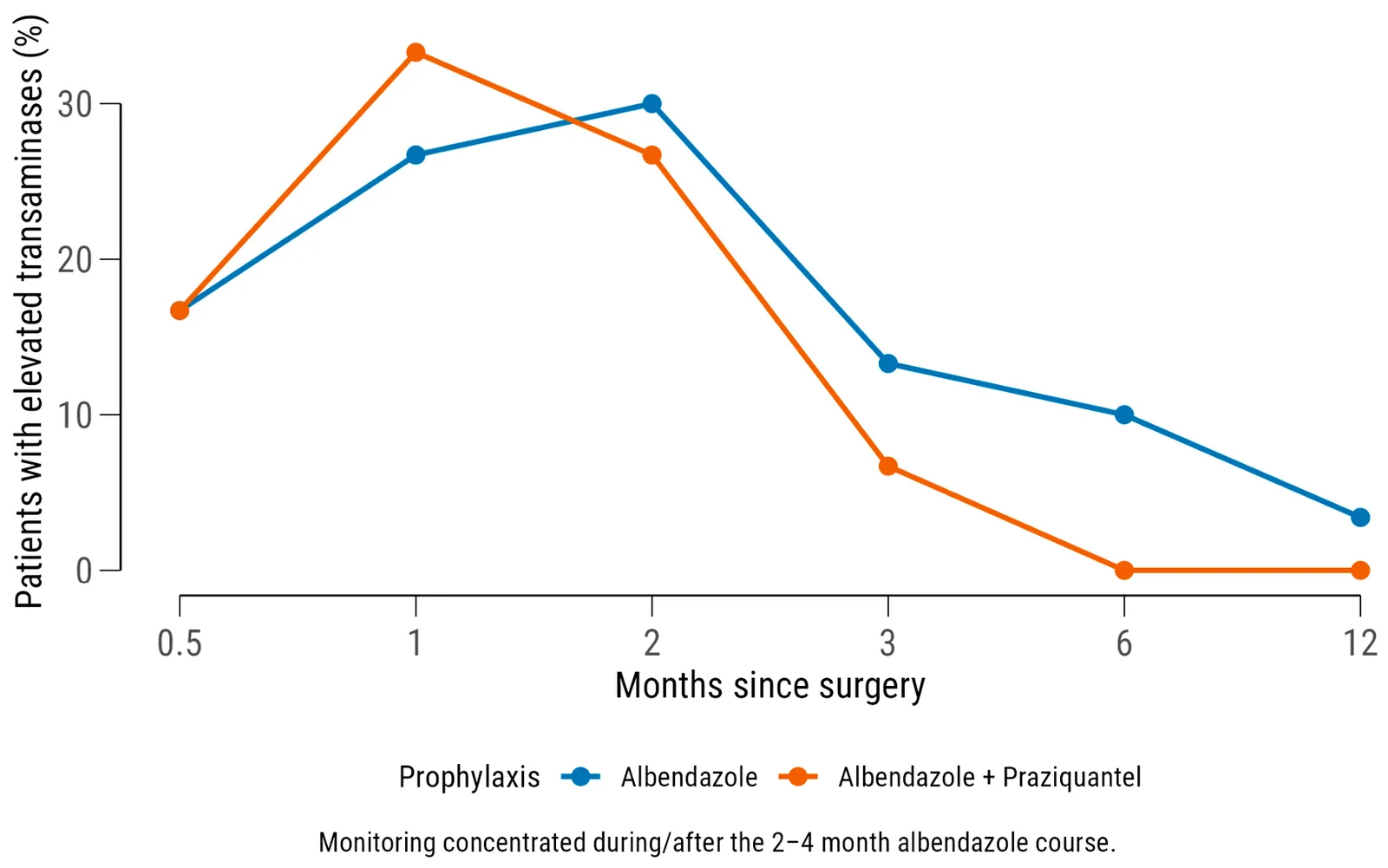
Proportion of patients with elevated transaminases (aspartate aminotransferase or alanine aminotransferase above the upper limit of normal of 40 U/L) at each scheduled monitoring visit, by prophylaxis arm (albendazole alone versus albendazole plus praziquantel). The *y*-axis shows the proportion of patients under observation at that visit with an elevation, and the *x*-axis shows months since surgery; denominators fall over time in line with the differential follow-up.

Longitudinal transaminase burden over the active treatment period (0–3 months), quantified as the incremental Area Under the Curve (AUC) above baseline, showed no significant differences between the treatment groups (Figure 4 and Figure 5). The median incremental AST AUC was 13.5 (IQR 4.1–28.9) U/L·month in the albendazole arm compared with 11.0 (IQR −3.8 to 20.3) U/L·month in the combination arm, with no statistically significant difference (Wilcoxon *p* = 0.325). Similarly, the baseline-adjusted linear model demonstrated no significant treatment effect (coefficient −1.07, *p* = 0.856). For ALT, the median incremental AUC was 33.4 (IQR 7.7–59.8) U/L·month in the albendazole arm and 7.2 (IQR −10.1 to 38.2) U/L·month in the combination arm. Although the unadjusted comparison approached statistical significance (Wilcoxon *p* = 0.060), the baseline-adjusted linear model again showed no significant difference between treatment groups (coefficient −5.23, *p* = 0.521).

A linear mixed model with a random patient intercept evaluating AST over 0.5–3 months demonstrated no significant main treatment effect (coefficient −0.04, 95% CI −5.60 to 5.52, *p* = 0.989) and no significant group-by-time interaction (coefficient −0.52, 95% CI −2.92 to 1.87, *p* = 0.666).

New-onset eosinophilia (defined as baseline eosinophils ≤ 5.0% rising to >5.0% on-treatment) occurred in two of 30 patients (6.7%) in the albendazole group and four of 30 patients (13.3%) in the combination group, with no significant difference between the arms (Fisher’s exact *p* = 0.671). All adverse events were transient, and all 60 patients successfully completed their prescribed prophylaxis course.

### 3.5. Disease Recurrence

**Figure 6:**
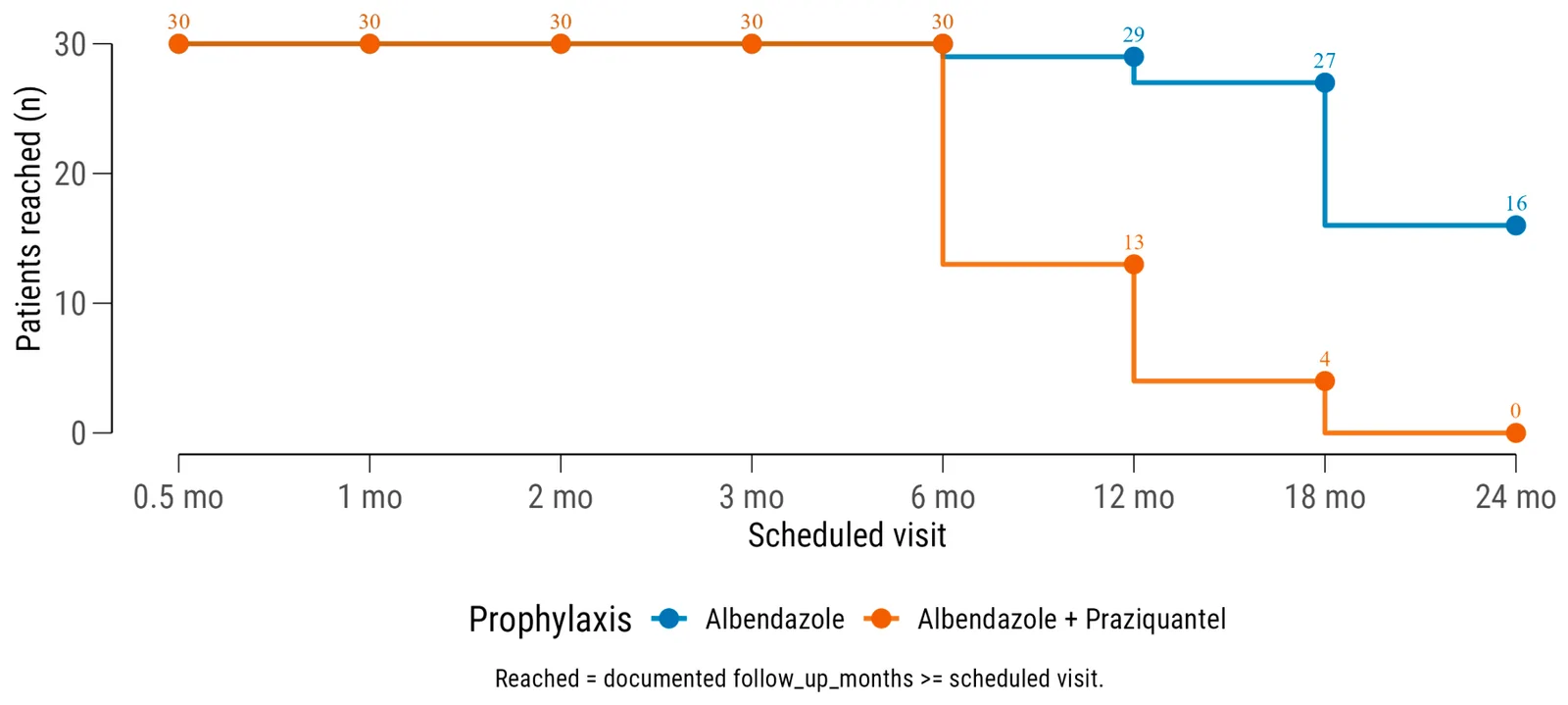
Per-visit denominators by prophylaxis arm. Reached = documented follow_up_months ≥ scheduled visit. Structural attrition in the combination arm is driven by the protocol follow-up window, not by random loss to follow-up.

The per-visit denominator trajectory makes the structural ascertainment context explicit: through the 6-month visit, all 60 patients were under active observation. From the 12-month visit onward, 29 of 30 albendazole patients and 13 of 30 combination patients reached the visit at which recurrence is first ascertainable. By 18 months, the combination arm denominator had fallen to 4 of 30, and by 24 months, no combination arm patient remained in observation (Figure 6). The attrition in the combination arm is structural (protocol-driven), not random, thereof every comparative inference from the 12-month landmark onward conditions on the surviving denominators shown.

Over the follow-up period, two recurrences of hepatic hydatidosis (6.7%) were observed in the albendazole group, whereas no recurrences (0.0%) were detected within the shorter follow-up available for the combination arm. The crude whole-cohort comparison of recurrence rates was not statistically significant (Fisher’s exact *p* = 0.492). Because crude proportions are confounded by the arms’ unequal observation time, person time incidence rates per 100 person-years were the design-appropriate summary: 3.73 (95% Poisson CI 0.45–13.48) recurrences per 100 person-years in the albendazole arm (2 events over 53.6 person-years) and 0.00 (95% Poisson CI 0.00–14.19) per 100 person-years in the combination arm (0 events over 26.0 person-years). To make the dependence on observation time explicit, recurrence incidence was additionally tabulated within follow-up strata (Supplementary File S1, Table S3b). Both recurrences accrued at the 12-month visit, so within the ascertainment interval (≥12 months) the albendazole arm contributed 24.1 person-years bearing both events (rate 8.30 per 100 person-years, 95% Poisson CI 1.01–30.00), whereas the combination arm contributed only 3.3 person-years and no events. In the pre-ascertainment interval (0–12 months) no recurrence is detectable in either arm by construction. The zero-recurrence count in the combination arm is therefore a direct consequence of its truncated post-12-month person time rather than of a demonstrably lower hazard.

As the primary efficacy comparison was anchored on the data overlap window (the 42-patient subcohort that reached the 12-month recurrence ascertainment landmark), the contrast was two of 29 (6.9%) in the albendazole arm versus zero of 13 (0.0%) in the combination arm. The corresponding risk difference was −6.9 percentage points (Newcombe 95% CI −22.0 to +16.5), and Fisher’s exact test in the restricted subcohort gave *p* = 1.000. The same separation pattern (zero events in the combination cell, two in the albendazole cell) is inherited, but with a transparent and smaller denominator that conditions only on patients whose endpoint could be ascertained at all.

**Figure 7:**
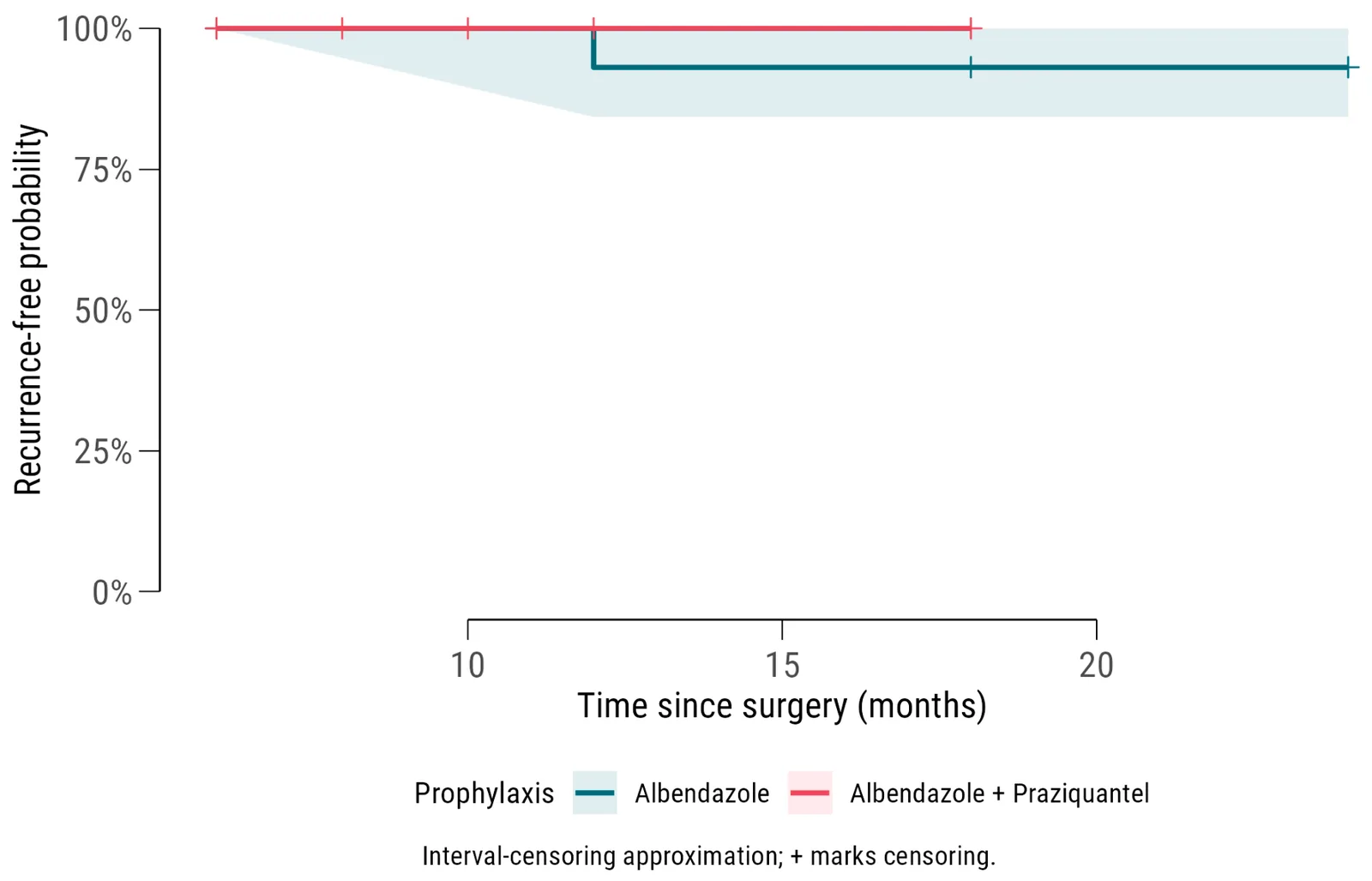
Kaplan–Meier estimates of recurrence-free survival by prophylaxis arm (albendazole alone versus albendazole plus praziquantel). Step functions show the probability of remaining recurrence-free (*y*-axis) against months since surgery (*x*-axis), with patients censored at their last documented recurrence assessment; tick marks denote censoring events. Both recurrences occurred in the albendazole arm at the 12-month visit. Because the combination arm was followed for a markedly shorter period and contributed no ascertained person time beyond early follow-up, the curves are not directly comparable, and the log-rank test (*p* = 0.338) is uninformative rather than evidence of equivalence.

Kaplan–Meier survival analysis of recurrence-free probability did not show a statistically significant difference between the groups (log-rank *p* = 0.338, chi-square = 0.919; Figure 7).

Expressed as absolute measures over the whole cohort, the between-arm risk difference was −6.7 percentage points (Newcombe 95% CI −21.3 to +5.7), and the corresponding risk ratio was 0.20 (Katz 95% CI 0.01 to 4.00). The latter is continuity-corrected and approximate because the combination arm contained no events and is correspondingly fragile. The recurrence probability derived as 1 − KM (equivalent to the cumulative incidence function in the absence of a competing risk variable) was 6.9% (95% CI 0.0–15.7%) in the albendazole arm at the 12-, 18-, and 24-month horizons and 0.0% in the combination arm, where the interval was not estimable owing to the absence of events (Supplementary File S1, Table S3).

**Figure 8:**
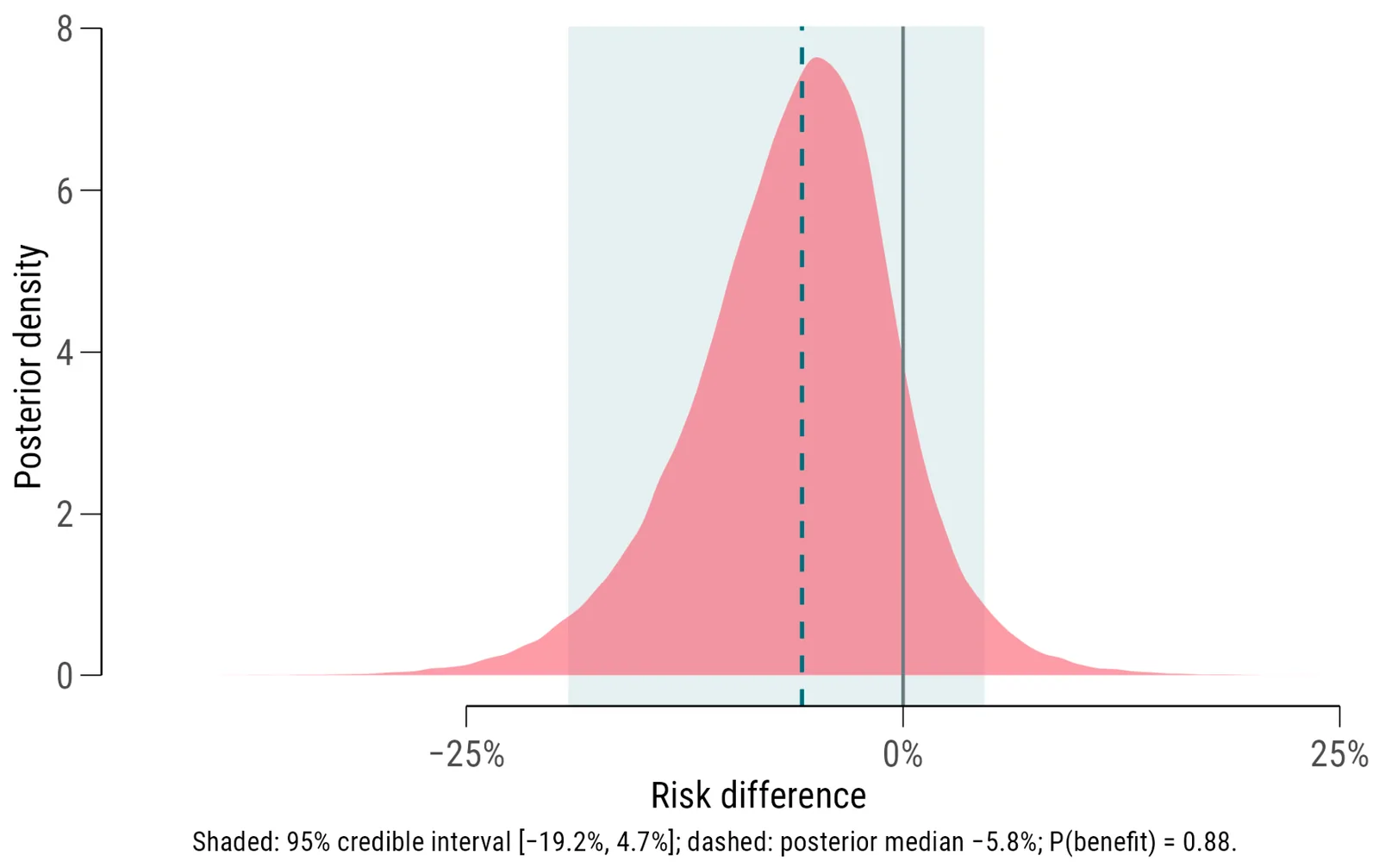
Posterior density of the recurrence risk difference (combination minus albendazole), derived from a conjugate Beta-Binomial analysis with a uniform Beta(1, 1) prior (200,000 Monte Carlo draws). Shaded band: 95% credible interval; dashed line: posterior median; solid vertical line: no-difference reference. The interval comfortably includes zero, and the posterior probability of benefit (0.88) lies below the conventional 0.95 threshold for moderate Bayesian evidence.

Because the zero-event combination cell renders the Fisher test uninformative and causes the frequentist time-to-event models to separate, the recurrence contrast was additionally summarized within a Bayesian conjugate Beta-Binomial framework. Under a uniform Beta(1, 1) prior, the posterior median event probability was 8.5% (95% credible interval [CrI] 2.0–21.4%) in the albendazole arm and 2.2% (95% CrI 0.1–11.2%) in the combination arm, giving a posterior risk difference of −5.8 percentage points (95% CrI −19.2% to +4.7%). The full posterior density is shown in Figure 8. The posterior probability that the combination conferred the lower recurrence risk was 0.88 under the uniform prior and 0.93 under the Jeffreys Beta (0.5, 0.5) prior, confirming robustness to the prior choice. Both values fall below the conventional 0.95 threshold for moderate Bayesian evidence (and well below the 0.975 threshold for strong evidence), and the 95% credible interval for the risk difference comfortably includes zero. For readers less familiar with Bayesian summaries, the posterior probability of benefit is the probability, conditional on the observed data and the prior, that the combination arm carries the lower true recurrence risk. Unlike a *p*-value it is a direct probability statement about the parameter, but a value of 0.88 still leaves roughly a one-in-eight posterior probability that the combination confers no benefit or harm, which is why it falls short of the 0.95 convention. The posterior probability is therefore a descriptive summary of a sparse, confounded, and differentially censored dataset, not evidence of efficacy. Thus, it must not be read either as demonstrating benefit or as establishing equivalence, and its principal value is that it remains finite and interpretable where the frequentist models separate. Consistent with this, a semi-parametric interval-censored proportional hazards model separated under the zero-event arm and yielded no usable hazard ratio.

A tipping point sensitivity analysis was conducted on the 18 unascertained patients (those with <12 months of follow-up; one in the albendazole arm and 17 in the combination arm). In the worst-case scenario where all unascertained patients are assumed to have recurred, the recurrence counts would change to 3 (10.0%) in the albendazole arm and 17 (56.7%) in the combination arm, which would yield a highly significant difference in the opposite direction (Pearson’s chi-square *p* < 0.001). This demonstrates that the observed absence of recurrence in the combination arm is highly sensitive to the shorter follow-up period, and that the apparent directional signal is contingent on the combination arm denominator’s premature truncation.

A detailed case-level analysis of the two recurrences observed in the albendazole group is presented below. Patient P27, a female treated for a single hepatic CE2 cyst (baseline size 120 mm) by partial pericystectomy, completed four months of postoperative albendazole therapy. At the 12-month follow-up, computed tomography demonstrated a new CE1 cyst within the surgical cavity, accompanied by a marked increase in anti-*Echinococcus* IgG titre from 53 (baseline) to 159 at 12 months. The patient was subsequently referred for repeat surgical intervention. Patient P35, also female, was treated for a single hepatic CE1 cyst (baseline size 96 mm) by partial pericystectomy and similarly completed four months of postoperative albendazole therapy. At the 12-month follow-up, computed tomography identified a new CE1 cyst within the postoperative cavity. This finding was accompanied by a progressive increase in anti-*Echinococcus* IgG titre from 76 at baseline to 90 at 3 months, 100 at 6 months, and 116 at 12 months. The patient remained under follow-up until 15 months and was ultimately referred for repeat surgical intervention.

## 4. Discussion

Cystic echinococcosis is characterized by a chronic clinical course and a notable propensity for postoperative recurrence, with the literature reporting recurrence rates between 2% and 22% [13,14,17,30,31,32]. Postoperative recurrences typically arise from several factors, including the presence of microcysts undetected during initial imaging or surgery, intraoperative spillage of viable cyst contents, or inadequate sterilization of the residual cavity [33,34]. Established predictive risk factors for disease recurrence include a history of prior interventions for CE, laparoscopic approaches, multiple cysts (three or more), large cysts, specific WHO ultrasound stages (e.g., CE1, CE2), and perioperative cyst rupture [35,36,37]. Consequently, meticulous surgical techniques to prevent intraoperative spillage and, where feasible, complete excision of the pericyst are crucial [13,38]. The viability of protoscolices within the hydatid fluid serves as a key determinant of recurrence risk. Local recurrence accounts for the vast majority (approximately 90%) of recurrent cases, defined as the development of new cysts within the surgical bed or residual cavity of the previously resected lesion [34].

Although recurrences occur after both radical and conservative surgical procedures [33,34,35], the rate of recurrence is significantly lower following radical resections [30,38]. When recurrence occurs, subsequent surgical interventions are substantially complicated by dense postoperative adhesions, which increase the risk of intraoperative complications and organ damage [34,35].

Differentiating postoperative fluid collections or residual cavities from early cyst recurrence remains a diagnostic challenge. Therefore, establishing a true recurrence requires a combination of serial imaging studies and longitudinal serological monitoring [14,34,39]. Rather than a single positive serological test, a documented rise in anti-*Echinococcus* IgG titres over time is highly indicative of active recurrence, as antibody titres can remain elevated for years following successful cyst eradication [13]. The serological trajectory of patient P27 in our cohort illustrates this principle directly: the anti-*Echinococcus* IgG titre increased threefold from the baseline at the 12-month visit coincident with imaging-confirmed recurrence. It is precisely the pattern expected of antigenic re-stimulation by a viable cyst and would have been less interpretable in the absence of serial sampling.

Albendazole is a benzimidazole derivative that has served as the cornerstone of medical therapy and chemoprophylaxis for human CE for several decades [18,40]. It induces structural degeneration of the germinal membrane, ultimately reducing or eliminating the viability of the hydatid cysts. The clinical efficacy of albendazole is mediated by its active metabolite, albendazole sulfoxide, whose absorption is highly lipophilic. Thus, co-administration with fatty meals significantly enhances its bioavailability and tissue penetration [41,42]. Long-term observational data from single-centre series support continuous benzimidazole exposure both perioperatively and as long-term suppressive therapy in inoperable or disseminated disease [38,43,44].

Current guidelines recommend starting albendazole 1 to 7 days preoperatively and continuing for 1 to 3 months postoperatively at a dose of 10–15 mg/kg/day (typically 400 mg twice daily in adults) to minimize the risk of secondary hydatidosis [6,15]. Practice surveys nonetheless reveal substantial heterogeneity in real-world use of perioperative chemotherapy, particularly regarding combination with praziquantel [8,9].

Despite standard albendazole prophylaxis, postoperative recurrences are still observed [34,45]. For instance, Prousalidis et al. [13] evaluated 51 patients with recurrent CE and noted similar recurrence rates between patients who received postoperative albendazole prophylaxis and those who did not. Our observation of recurrences in the albendazole monotherapy group further supports the view that albendazole alone does not guarantee complete protection against recurrence.

To address these limitations, several clinical and experimental studies have evaluated the combination of albendazole and praziquantel, particularly to prevent secondary dissemination following intraoperative spillage [17,19,23,28,46]. Praziquantel, a pyrazinoisoquinolone derivative, has direct protoscolicidal effects and possesses a favourable safety profile [19,47]. Pharmacokinetic studies demonstrate that co-administering praziquantel increases the Area Under the Curve (AUC) of the active metabolite albendazole sulfoxide by approximately 4.5-fold without modifying praziquantel pharmacokinetics or increasing adverse event rates. When taken with a high-fat meal, the concentration increases up to 12-fold [19,48,49]. This synergistic interaction leads to elevated concentrations of albendazole sulfoxide in both serum and cyst fluid, resulting in greater ultrastructural damage to the germinal layer and protoscolices [28,41], though some authors have reported more variable findings regarding intracystic drug levels [48].

However, clinical data regarding the efficacy of combined albendazole and praziquantel regimens in preventing human CE recurrence remain limited [16,19]. In a cohort study by Dautović-Krkić et al. [50], 30 of 136 surgically treated patients received a 14-day course of postoperative praziquantel (25–50 mg/kg/day) combined with corticosteroids, resulting in zero recurrences over a 1.5- to 5.5-year follow-up period, compared to a 12.5% recurrence rate in the surgery-only group. Similarly, Cobo et al. [28] reported that preoperative combination therapy for one month led to complete protoscolex non-viability in 90.4% of patients with abdominal cysts, compared with 75% and 57.1% of patients receiving albendazole monotherapy at 10 mg/kg/day and 20 mg/kg/day, respectively. Ayles et al. [17] also observed that only one of 25 patients receiving combined preoperative therapy (albendazole and praziquantel) harboured viable protoscolices at the time of surgery, compared to five of eight patients who received albendazole alone. A 20-year retrospective single-centre experience similarly reported a more favourable parasitological response in patients receiving combination chemotherapy than in those receiving benzimidazole monotherapy [51], Combination therapy has also been used effectively in case-level salvage scenarios such as disseminated and recurrent brain disease [52,53], underlining its potential value when monotherapy proves insufficient.

Establishing an optimal dosage regimen for combined therapy remains challenging due to the heterogeneity of protocols used across historical studies [19,54]. The latest WHO guidelines suggest a two-week postoperative course of praziquantel (40–50 mg/kg/day) in addition to albendazole in cases of intraoperative cyst spillage [15]. The dose of praziquantel used in our study (25 mg/kg/day for 10 days) was lower than this contemporary recommendation and was shorter in duration, as the institutional protocol predated the current guidance and was designed based on the historical regimen of Cobo et al. [28]. Two implications follow. First, the regimen tested here is not directly comparable to current best-practice combination chemoprophylaxis, and the directional Bayesian signal observed should not be extrapolated to the higher contemporary dose without prospective evaluation. Whether a higher praziquantel dose would yield additional efficacy at the cost of additional toxicity remains an open question that the present cohort is not powered to address. Second, the safety findings from a lower-dose regimen represent a lower bound on the hepatotoxicity that may be encountered under the current WHO-recommended dose, since dose-dependent adverse event rates above 40 mg/kg/day have been documented previously [19,55]. Future prospective studies should therefore evaluate the currently recommended dose explicitly, both for efficacy and for safety.

Regarding safety, albendazole is generally well tolerated. Reversible elevation of liver transaminases is the most common adverse effect, occurring in approximately 10–20% of cases. Other potential side effects include gastrointestinal distress, alopecia, headache, and rare myelosuppression [56,57,58]. Praziquantel is also associated with mild, dose-dependent side effects such as abdominal pain, dizziness, and headache, particularly at doses exceeding 40 mg/kg/day [19,55]. The combined regimen of albendazole and praziquantel has demonstrated a favourable safety profile, with adverse effects generally being mild and self-limiting, as evidenced by pharmacovigilance reviews and clinical cohort studies [55,59]. In our cohort, the incidence and severity of adverse events did not differ significantly between the two groups, with transient transaminase elevation being the predominant finding. Under the a priori DAG-aligned adjustment set (baseline AST plus age, identified as the minimal sufficient adjustment set for the total effect of treatment on hepatotoxicity), the adjusted odds of elevated transaminases in the combination arm were 1.45 (95% CI 0.49–4.37). The sensitivity {AST, ALT} and {AST, ALT, age} specifications gave directionally identical estimates (OR 1.40 and 1.48, respectively), as did the PS-adjusted model (OR 1.60). The corresponding E-value for the point estimate was 2.26, because the confidence interval for the adjusted odds ratio already includes the null, no unmeasured confounding is required for compatibility with the absence of added hepatotoxicity, and an E-value for the confidence limit is therefore not interpretable as evidence of robustness and is not reported. The safety conclusion rests on the confidence interval spanning the null across the DAG-aligned, sensitivity, and propensity score specifications rather than on E-value robustness. Taken together, the safety analyses provide no convincing additional hepatotoxicity signal attributable to praziquantel, consistent with the safety profile reported in the international literature.

Drug-induced eosinophilia is a recognized phenomenon, although it is not typically attributed to albendazole monotherapy. Rather, eosinophilia observed during the initiation of albendazole treatment is generally interpreted as a host immune response to antigen release from dying cysts [3]. However, in our study, since the drug was administered postoperatively following complete cyst removal, the observed eosinophilia could point to minimal spillage of hydatid fluid during the surgical intervention, the presence of subclinical, undetected small cysts, or transient hypersensitivity reactions, as none of the patients had a history of atopy, allergies, or concomitant drug use.

Given that recurrences of hydatid disease may occur late, often between 3 and 10 years after intervention, long-term follow-up of at least 5 years is strongly recommended [11,14]. Active post-treatment surveillance using serial imaging and serology is vital for the early detection of asymptomatic recurrences [31,35,60].

## 5. Strengths and Limitations

Several limitations, intrinsic to the design, constrain the inferences that can be drawn and warrant explicit statement.

First, the study is observational and retrospective: allocation to the two regimens was not randomized but determined by clinical judgement and by the contraindications to praziquantel, so the comparison is subject to confounding by indication, and the retrospective abstraction of routinely collected records introduces the potential for selection and information bias. The consequence of non-randomization was directly observable in the data: 14 of the 15 measured baseline covariates exceeded the conventional |SMD| > 0.10 balance threshold (Supplementary File S1, Table S1 and Figure S1), indicating that the two arms were not exchangeable at baseline and that any crude between-arm contrast is confounded. The single quantitatively balanced covariate was the per-patient hepatic cyst count. Conversely, the most extreme cyst-level imbalance was the abdominal cyst burden, which was carried almost entirely by the combination arm (22 of 24 abdominal cysts) and was concentrated in two patients with disseminated intra-abdominal disease. Critically, allocation was governed by clinical judgement, and the combination arm systematically comprised patients with more complex or higher-burden disease (more abdominal and fertile cysts, larger baseline cyst size, and higher baseline transaminases). The confounding by indication therefore ran toward assigning worse-prognosis patients to the combination regimen, which would, if anything, bias the recurrence contrast against the combination, but the magnitude of this bias cannot be quantified from these data. Although standardized mean differences, propensity score and inverse probability weighting, and the DAG-aligned baseline-adjusted model with its accompanying E-value were used to mitigate these imbalances for the adequately powered safety endpoints, the limited region of common support means residual confounding cannot be excluded, and the adjusted safety estimates remain observational rather than causal.

Second, follow-up duration differed systematically between the arms, with the combination-treated patients followed for a markedly shorter period (median 10 versus 24 months). This differential, protocol-driven follow-up is the central analytic limitation, producing structural (non-random) missingness in later visits and rendering the apparent absence of recurrence in the combination arm strongly dependent on the shorter observation window. The tipping point analysis makes this dependence quantitative and should be read as the decisive constraint on interpretation: if the 17 combination arm patients with less than 12 months of follow-up were assumed to have recurred, the direction of the contrast would reverse and become highly significant in favour of monotherapy (Figure 6). The observed directional signal is therefore fully contingent on the unobserved outcomes of prematurely censored patients.

Third, the recurrence endpoint accrued only two events, both in the monotherapy arm. This complete separation renders frequentist hypothesis tests and time-to-event models uninformative, and although the Bayesian re-analysis provides a principled, finite summary that favours the combination directionally (posterior probability 0.88 under the uniform prior and 0.93 under the Jeffreys prior), both values fall below the conventional 0.95 threshold for moderate Bayesian evidence and the 95% credible interval for the risk difference includes zero. Taken together, the combination of non-randomized allocation, severe baseline imbalance, structural differential follow-up, and accrual of only two events means that no causal inference about the comparative efficacy of the combination regimen for recurrence prevention can be drawn from these data. Therefore, the directional Bayesian signal is hypothesis-generating-only.

Fourth, the cohort was drawn from a single centre and comprising a convenience sample of 60 patients without a formal a priori sample size calculation, limiting both power and generalizability. Fifth, the praziquantel dose used (25 mg/kg/day for 10 days) was lower and shorter than the current WHO-recommended post-spillage regimen (40–50 mg/kg/day for 14 days), so the findings address a historical rather than a contemporary regimen.

Finally, the follow-up period was relatively short relative to the natural history of cystic echinococcosis, in which recurrences can manifest between three and 10 years after intervention [11,14,31]. The observation window must be extended to at least five years before long-term outcomes can be assessed.

The principal strengths of the study are the prespecified and transparent analytic strategy (Supplementary File S2), the use of design-appropriate balance metrics, per-visit denominator tabulation, and absolute effect measures in place of reliance on significance thresholds, the a priori DAG-based specification of adjustment sets, the routine reporting of credible interval inclusion of the null and of the E-value, and the explicit, rather than concealed, treatment of the design’s structural limitations.

## 6. Challenges and Future Perspectives

Several challenges continue to constrain the evaluation of combination chemoprophylaxis for cystic echinococcosis and frame the agenda for future work. The first is the rarity and long latency of the outcome: postoperative recurrence is uncommon and can appear three to 10 years after surgery, so any single-centre cohort accrues few events within a feasible follow-up window and is structurally underpowered for an efficacy contrast [14,31]. The second is the persistent heterogeneity of regimens, with praziquantel dose (25 to 50 mg/kg/day), dosing frequency, and duration varying widely across historical and contemporary protocols, which frustrates pooling and cross-study comparison [15,19,54]. The third is the difficulty of distinguishing true recurrence from residual postoperative cavities and of standardizing viability assessment, since microscopy is available for only a minority of resected cysts and serology remains positive for years after successful treatment [13,34]. The fourth is the near-absence of randomized evidence: combination chemoprophylaxis has been examined almost exclusively in observational studies that are subject to confounding by indication and differential follow-up, which are the same limitations that constrain the present cohort [16,24].

These challenges define the future agenda. A properly powered, prospective, multicentre randomized controlled trial with balanced and prolonged follow-up of at least five years is required to test whether the combination confers a clinically meaningful reduction in recurrence, and such a trial should adopt the currently WHO-recommended praziquantel dose and evaluate efficacy and hepatotoxicity jointly, since the safety findings reported here derive from a lower-dose regimen and represent a lower bound on the toxicity expected at contemporary doses [15,55]. Standardized definitions of recurrence, viability, and cavity obliteration, together with harmonized imaging and serological follow-up schedules, would improve comparability across centres, and emerging diagnostic technologies such as nanobiosensors may sharpen the early detection of viable residual disease [10]. Until such evidence is generated, the role of adding praziquantel to postoperative albendazole should be regarded as an open question supported by mechanistic and experimental rationale but not yet by robust clinical outcome data.

## 7. Conclusions

The concomitant administration of albendazole and praziquantel was well tolerated, with no convincing additional hepatotoxicity signal once baseline enzyme differences and the a priori DAG-aligned adjustment set were accounted for. Across every adjusted specification the confidence interval for elevated transaminases included the null, indicating no detectable added hepatotoxicity rather than a robustly estimated effect. Given the non-randomized allocation, the severe baseline imbalance, the structural differential follow-up (whose impact the tipping point analysis shows to be decisive), and the accrual of only two recurrence events, these data cannot support any conclusion about the comparative efficacy of the combination regimen for recurrence prevention, and efficacy cannot be reliably assessed in this cohort. The directional Bayesian signal (posterior probability of benefit 0.88 under the uniform prior, 0.93 under the Jeffreys prior, both below the conventional 0.95 threshold for moderate Bayesian evidence) is hypothesis-generating-only and must not be interpreted as evidence of superiority. The combination of albendazole and praziquantel remains a candidate intervention worth evaluating prospectively, and the findings support the conduct of an adequately powered, prospective, multicentre randomized controlled trial with balanced follow-up of at least five years and, ideally, the currently WHO-recommended praziquantel dose, before any recommendation for routine clinical practice can be made.

## Figures and Tables

**Table 1:** Baseline demographic and clinical characteristics of the study cohort.

Parameter	Albendazole (n = 30)	Albendazole + Praziquantel (n = 30)	*p*
Age (years, mean ± SD)	47.0 ± 15.0	40.8 ± 14.9	0.114
Sex (female/male, n [%])	16 (53.3%)/14 (46.7%)	14 (46.7%)/16 (53.3%)	0.606
Residence (city/village, n [%])	20 (66.7%)/10 (33.3%)	13 (43.3%)/17 (56.7%)	0.069
Primary diagnosis (n [%])			0.674
Hepatic	22 (73.3%)	19 (63.3%)	
Pulmonary	5 (16.7%)	6 (20.0%)	
Hepatic and pulmonary	1 (3.3%)	3 (10.0%)	
Hepatic and abdominal	1 (3.3%)	0 (0.0%)	
Abdominal	1 (3.3%)	2 (6.7%)	
Albendazole duration (months, median [IQR])	3.0 [3.0–4.0]	3.0 [3.0–4.0]	0.336
follow-up duration ^1^ (months, median [range])	24 [6–24]	10 [6–18]	

^1^ Follow-up duration differs systematically between arms due to the protocol’s staggered enrollment design; statistical comparison is therefore omitted. Abbreviations: SD, standard deviation; IQR, interquartile range; n, number of patients.

**Table 2:** Characteristics of surgically removed hydatid cysts summarized at the per-patient and cyst level.

Parameter	Albendazole (n = 30)	Albendazole + Praziquantel (n = 30)	*p*
Per patient			
Cysts per patient (median [IQR])	1.0 [1.0–2.0]	1.0 [1.0–3.0]	0.238
Mean ± SD (range)	1.6 ± 1.0 (1–5)	2.4 ± 2.6 (1–12)	
Largest cyst per patient (mm, Median [IQR])	93.0 [60.8–119.0]	100.0 [80.0–127.5]	0.236
Mean ± SD (range)	92.5 ± 45.9 (30–250)	112.8 ± 59.6 (35–300)	
Largest cyst ≤ 100 mm, n (%)	20 (66.7%)	18 (60.0%)	0.592
Largest cyst > 100 mm, n (%)	10 (33.3%)	12 (40.0%)	
Cyst-level, by anatomical site (n [% of arm’s cysts])			
Hepatic	35 (74.5%)	36 (50.7%)	
Pulmonary	10 (21.3%)	13 (18.3%)	
Abdominal	2 (4.3%)	22 (31.0%)	
Cyst-level, by maximum diameter (n [% of arm’s cysts])			0.547 ^1^
≤100 mm	30 (63.8%)	50 (70.4%)	
>100 mm	17 (36.2%)	21 (29.6%)	

^1^ Fisher’s exact test (OR for >100 mm in combination vs. albendazole 0.74, 95% CI 0.32–1.76). Cyst-level tests assume independence between cysts; this assumption is approximate since a small number of patients contribute multiple cysts (see Supplementary File S1).

**Table 3:** Operative techniques for liver hydatidosis.

Operative Technique	Albendazole (n = 24)	Albendazole + Praziquantel (n = 22)
Partial pericystectomy	16 (66.7%)	14 (63.6%)
Cystopericystectomy	6 (25.0%)	5 (22.7%)
Segmentectomy	0 (0.0%)	2 (9.1%)
Lobectomy	1 (4.2%)	1 (4.5%)
Partial + cystopericystectomy	1 (4.2%)	0 (0.0%)

Values are n (%) of patients with a hepatic component (24 in the albendazole arm, 22 in the combination arm). No statistically significant difference in technique distribution was observed (Fisher’s exact *p* = 0.722). n, number of patients.

**Table 4:** Microscopic examination of hydatid fluid samples.

Parameter	Albendazole	Albendazole + Praziquantel
Examined samples (n)	7	14
Positive samples (n [%])	5 (71.4%)	9 (64.3%)

Microscopic examination of hydatid fluid was available for only 21 of the 118 resected cysts (7 in the albendazole arm, 14 in the combination arm). Percentages refer to examined samples only and cannot be generalized to all resected cysts. The patient-level fertility variable used elsewhere in the manuscript is a proxy combining this partial microscopy with histological notes, not a complete cyst-level assay.

**Table 5:** Adverse events reported during postoperative prophylaxis.

Adverse Event ^1^	Albendazole (n = 30)	Albendazole + Praziquantel (n = 30)
Elevated transaminases (worst grade)	11 (36.7%)	13 (43.3%)
≤2 × ULN	6 (20.0%)	9 (30.0%)
>2–3 × ULN	5 (16.7%)	4 (13.3%)
Anemia (Hb 103–113 g/L)	1 (3.3%)	1 (3.3%)
Abdominal discomfort	1 (3.3%)	2 (6.7%)
Vomiting	0 (0.0%)	1 (3.3%)
Any adverse event (patients)	13 (43.3%)	15 (50.0%)

^1^ Individual event counts do not sum to the patient totals because some patients experienced multiple concurrent adverse events. ULN = upper limit of normal (40 U/L for both AST and ALT).

## Data Availability

The individual patient-level data are not publicly available. This is a single-centre cohort of 60 patients with a rare disease, which cannot be robustly de-identified against re-identification. Their release would be incompatible with patient confidentiality and the terms of the institutional ethics approval. The data are available from the corresponding author on reasonable request, subject to ethics approval and a data sharing agreement. To enable full computational reproducibility of the analytic workflow without disclosing any patient record, the complete annotated R script that produces every table and figure in the article and in Supplementary File S1 is provided as Supplementary File S2 (analysis.R), together with a complete data dictionary (documenting every variable, its unit, range, and missingness) and a synthetic surrogate dataset of identical structure. The synthetic dataset is generated by random draws from the documented variable ranges and factor levels; it contains no real patient information and does not reproduce the study results, but it allows the entire pipeline to be executed end to end so that the code can be independently verified. On the real data, held by the authors, the same script reproduces every reported result. The completed STROBE checklist for cohort studies, with page references into the present manuscript, is provided as Supplementary File S3.

## Supplementary Materials

The following supporting information can be downloaded at: https://www.mdpi.com/article/10.3390/biomedicines14081734/s1. Supplementary File S1 (methodological diagnostics, sensitivity analyses, and balance and missingness assessments) contains: Table S1: Baseline covariate balance between prophylaxis arms across all fifteen measured covariates, with standardized mean differences; Figure S1: Love plot of baseline covariate standardized mean differences (albendazole minus combination); Table S2: Absolute between-arm effect measures (risk differences and risk ratios) across the full binary endpoint set; Table S3a: Fixed horizon recurrence probability at 12, 18, and 24 months, derived as 1 − Kaplan–Meier; Table S3b: Recurrence incidence stratified by follow-up interval (person time), by prophylaxis arm; Figure S2: Distribution of follow-up duration by prophylaxis arm; Table S4: Per-visit denominators by prophylaxis arm; Figure S3: Propensity score overlap diagnostic and region of common support; Table S5: Standardized mean differences before versus after inverse probability of treatment weighting; Figure S4: Absolute standardized mean differences before and after IPW (weighted Love plot); Figure S5: Missingness map by analyte family, scheduled visit, and arm; Figure S6: Directed acyclic graph defining the a priori adjustment set for the hepatotoxicity endpoint. Supplementary File S2: Complete annotated R analysis script (‘analysis.R’) reproducing every table and figure in the main article and in Supplementary File S1, accompanied by a data dictionary and a synthetic surrogate dataset that allows the analysis to be executed without disclosing any patient record. Supplementary File S3: STROBE checklist for cohort studies.

## Abbreviations

The following abbreviations are used in this manuscript:

ALT	Alanine Aminotransferase
AST	Aspartate Aminotransferase
ATE	Average Treatment Effect
AUC	Area Under the Curve
CE	Cystic Echinococcosis
CI	Confidence Interval
CrI	Credible Interval
CT	Computed Tomography
DAG	Directed Acyclic Graph
ELISA	Enzyme-Linked Immunosorbent Assay
EPV	Events Per Variable
Hb/Hgb	Hemoglobin
IgG	Immunoglobuline G
IPW	Inverse Probability of Treatment Weighting
IQR	Interquartile Range
IWGE	Informal Working Group on Echinococcosis
KM	Kaplan–Meier
LMM	Linear Mixed Effects Model
OR	Odds Ratio
PAIR	Puncture, Aspiration, Injection, Re-Aspiration
PS	Propensity Score
RD	Risk Difference
RR	Risk Ratio
SD	Standard Deviation
SE	Standard Error
SMD	Standardized Mean Difference
STROBE	Strengthening the Reporting of Observational Studies in Epidemiology
ULN	Upper Limit of Normal
WHO	World Health Organization
