# Supplementary material for: Postoperative Prophylaxis of Cystic Echinococcosis with Albendazole and Praziquantel: A Retrospective Cohort Study
Source: Biomedicines. 2026 Jul 31;14(8):1734. doi: 10.3390/biomedicines14081734 (PMC13510079; doi:10.3390/biomedicines14081734)
Supplement: Supplementary file 1 [file biomedicines-14-01734-s001.zip › Supplementary File S1.pdf]

biomedicines

MDPI

Supplementary File S1: Additional Tables and Figures

Postoperative Prophylaxis of Cystic Echinococcosis with Albendazole and Praziquantel: A Retrospective Observational Cohort Study

Galya Popova-Daskalova[1,6,*], Kostadin Kostadinov[2,3], Krasimira Eneva[1,6], Albena Masarlieva[1,6], Dimitar Vuchev[1], Angel Uchikov[4,6], Lyubka Aleksova[4,6], Nikolay Nikolov[4,6], Tanya Nedkova[5]

[1] Department of Infectious Diseases, Parasitology and Tropical Medicine, Section of Parasitology, Medical University of Plovdiv, Bulgaria, galiya.popova@mu-plovdiv.bg, krasimira.eneva@mu-plovdiv.bg, albena.masarlieva@mu-plovdiv.bg, dimitar.vuchev@mu-plovdiv.bg
[2] Department of Social Medicine and Public Health, Medical University of Plovdiv, 4002 Plovdiv, Bulgaria, kostadinr.kostadinov@mu-plovdiv.bg
[3] Environmental Health Division, Research Institute at Medical University of Plovdiv, 15-A "Vasil Aprilov" Blvd, 4002 Plovdiv, Bulgaria
[4] Department of Special Surgery, Medical University of Plovdiv, Bulgaria, angel.uchikov@mu-plovdiv.bg, lyubka.aleksova@mu-plovdiv.bg, nikolay.st.nikolov@mu-plovdiv.bg
[5] Faculty of Medicine, Medical University of Plovdiv, Bulgaria, tanyanedkova26@gmail.com
[6] "St. George" University Hospital, Plovdiv, Bulgaria
*Correspondence: galiya.popova@mu-plovdiv.bg

This supplementary file collects the methodological diagnostics, sensitivity analyses, and detailed balance and missingness assessments that support the main article but would interrupt its narrative if reproduced in the body. All tables and figures were generated by the same single annotated R script that produced the main-article output, supplied as Supplementary File 2.

The supplementary content is organised in the order in which it is first cited from the main article: baseline covariate balance (Section S1), absolute effect measures across endpoints (Section S2), fixed-horizon recurrence probability (Section S3), differential follow-up distribution and per-visit denominators (Section S4), propensity-score overlap diagnostics (Section S5), post-weighting covariate balance (Section S6), structural-versus-sporadic missingness (Section S7), and the directed acyclic graph that defines the a priori adjustment set for the hepatotoxicity endpoint (Section S8). Section S9 provides the full technical specification of the statistical procedures, relocated from the main article at the reviewers' request to improve readability. The full posterior risk-difference density for the recurrence endpoint, previously presented in this supplement, is now reported in the main article as Figure 8.

S1. Baseline Covariate Balance under Non-randomized Allocation

Because allocation was non-randomized, baseline covariate balance was assessed using standardized mean differences (SMDs), with |SMD| > 0.10 flagged as imbalance regardless of the accompanying significance test. The full balance table (Table S1) covers all fifteen baseline covariates: eleven continuous (age, total cyst count, the three per-organ cyst counts, baseline largest-cyst diameter, baseline serology titre, baseline haemoglobin,

baseline eosinophils, baseline AST, baseline ALT) and four binary (female sex, village residence, hepatic-only diagnosis, fertile cyst). The Love plot (Figure S1) provides the corresponding visual summary.

Table S1. Baseline covariate balance between prophylaxis arms (all 15 measured covariates).

Covariate	Type	Albendazole (n = 30)	Albendazole + Praziquantel (n = 30)	p	SMD	Balance flag
Age (years)	Continuous (mean ± SD)	47.0 ± 15.0	40.8 ± 14.9	0.114	0.415	Imbalanced
Number of cysts	Continuous (mean ± SD)	1.6 ± 1.0	2.4 ± 2.6	0.238	−0.414	Imbalanced
Hepatic cysts (n)	Continuous (mean ± SD)	1.2 ± 0.9	1.2 ± 1.1	0.829	−0.033	Balanced
Pulmonary cysts (n)	Continuous (mean ± SD)	0.3 ± 1.0	0.4 ± 0.8	0.353	−0.115	Imbalanced
Abdominal cysts (n) †	Continuous (mean ± SD)	0.1 ± 0.3	0.7 ± 2.8	0.959	−0.335	Imbalanced
Cyst size at baseline (mm)	Continuous (mean ± SD)	92.5 ± 45.9	112.8 ± 59.6	0.236	−0.381	Imbalanced
Baseline serology titre	Continuous (mean ± SD)	55.1 ± 58.7	81.4 ± 106.5	0.359	−0.305	Imbalanced
Baseline haemoglobin (g/L)	Continuous (mean ± SD)	136.1 ± 15.5	133.4 ± 14.5	0.520	0.182	Imbalanced
Baseline eosinophils (%)	Continuous (mean ± SD)	3.2 ± 2.4	2.9 ± 1.4	0.941	0.158	Imbalanced
Baseline AST (U/L)	Continuous (mean ± SD)	24.6 ± 9.7	31.7 ± 18.5	0.096	−0.478	Imbalanced
Baseline ALT (U/L)	Continuous (mean ± SD)	28.3 ± 25.2	37.6 ± 26.9	0.018	−0.357	Imbalanced
Female sex	Binary, n (%)	16 (53.3%)	14 (46.7%)	0.606	0.134	Imbalanced
Village residence	Binary, n (%)	10 (33.3%)	17 (56.7%)	0.069	−0.482	Imbalanced
Hepatic-only diagnosis	Binary, n (%)	22 (73.3%)	19 (63.3%)	0.405	0.216	Imbalanced
Fertile cyst	Binary, n (%)	16 (53.3%)	21 (70.0%)	0.184	−0.348	Imbalanced

Note: SMD = standardized mean difference (albendazole minus combination); the convention treats |SMD| > 0.10 as imbalance. † The abdominal-cysts distribution is heavily right-skewed (zero cysts in 56 patients; 1, 1, 10 and 12 in the remaining four), so the mean-based SMD is unstable; the substantive cyst-level signal is reported in the main article's cyst-characteristics table. Fourteen of 15 covariates exceed the |SMD| > 0.10 threshold; the per-patient hepatic cyst count is the single balanced covariate.

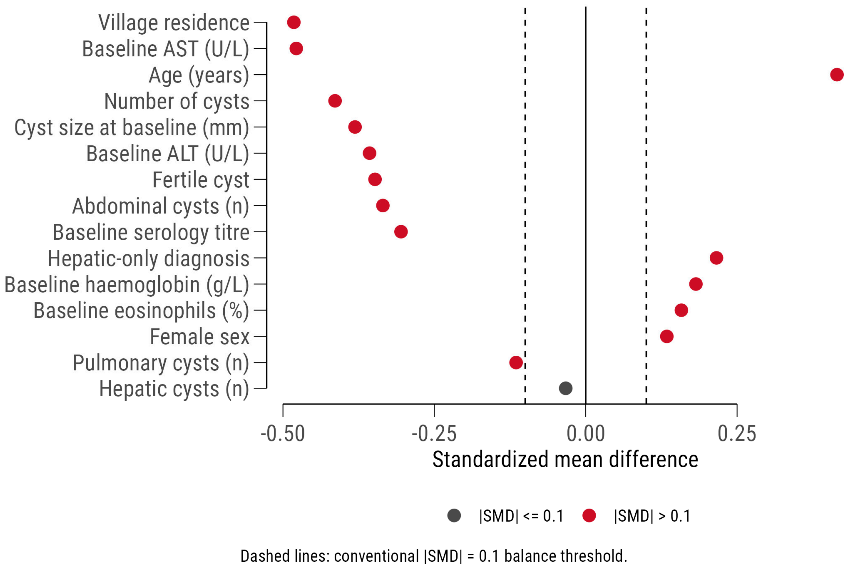

Figure S1. Baseline covariate standardized mean differences (albendazole minus combination). Dashed lines represent the conventional balance threshold of 0.10.

S2. Absolute Effect Measures across the Endpoint Set

Crude tests reported in the main article are complemented here by absolute between-arm contrasts (Table S2). The risk difference (RD) is reported in percentage points using the Newcombe hybrid-score interval [1]; the risk ratio (RR) is reported using the Katz logarithmic interval, with a 0.5 continuity correction applied when an arm has zero events. The convention throughout is combination versus albendazole (RD = combination − albendazole; RR = combination ÷ albendazole), matching the contrast direction used in the Bayesian re-analysis.

Table S2. Absolute effect measures (risk difference and risk ratio) for all binary endpoints, combination versus albendazole.

Endpoint	Albendazole (events / *n*)	Albendazole + Praziquantel (events / *n*)	Risk difference (pp) [95% CI]	Risk ratio [95% CI]
Recurrence (ever)	2 / 30 (6.7%)	0 / 30 (0.0%)	−6.7 [−21.3, 5.7]	0.20 [0.01, 4.00] *
Any side effect	13 / 30 (43.3%)	15 / 30 (50.0%)	+6.7 [−17.6, 29.9]	1.15 [0.67, 1.99]
Elevated transaminases	11 / 30 (36.7%)	13 / 30 (43.3%)	+6.7 [−17.3, 29.6]	1.18 [0.63, 2.20]
Anaemia	1 / 30 (3.3%)	1 / 30 (3.3%)	+0.0 [−13.6, 13.6]	1.00 [0.07, 15.26]
Abdominal discomfort	1 / 30 (3.3%)	2 / 30 (6.7%)	+3.3 [−10.8, 18.2]	2.00 [0.19, 20.90]
Vomiting	0 / 30 (0.0%)	1 / 30 (3.3%)	+3.3 [−8.3, 16.7]	not estimable

Note: pp = percentage points. RD intervals use the Newcombe hybrid-score method; RR intervals use the Katz log method. The asterisk on the recurrence risk ratio marks a continuity-corrected, approximate estimate (combination arm contained no events); this estimate is fragile and should not anchor interpretation. The Bayesian re-analysis in the main article supplies the principled summary of the 2-vs-0 contrast.

S3. Fixed-horizon Recurrence Probability (1 − KM)

Because recurrence was ascertained only at the scheduled 12-, 18- and 24-month visits, the endpoint is interval-censored. With no competing-risk variable in the dataset, the cumulative incidence function reduces to 1 − KM (it is therefore not an Aalen–Johansen estimator). With only two events the resulting horizon probabilities carry wide intervals and are reported in Table S3 for clinical readability only.

Table S3a. Fixed-horizon recurrence probability at 12, 18 and 24 months, derived as 1 − KM.

Arm	Horizon (months)	Recurrence probability (1 − KM)	95% CI
Albendazole	12	6.9%	[0.0%, 15.7%]
Albendazole	18	6.9%	[0.0%, 15.7%]
Albendazole	24	6.9%	[0.0%, 15.7%]
Albendazole + Praziquantel	12	0.0%	not estimable (0 events)
Albendazole + Praziquantel	18	0.0%	not estimable (0 events)
Albendazole + Praziquantel	24	0.0%	not estimable (0 events)

Note: the 95% CI is obtained by inverting the Greenwood-variance KM survival CI; the combination-arm CI is not estimable because the standard error of the Kaplan-Meier estimate degenerates under zero observed events. The horizon estimate in the albendazole arm is constant from 12 months onward because both events occurred at the 12-month visit, and no additional events were recorded by the 24-month horizon.

To quantify the impact of the differential follow-up directly on the person-time scale, recurrence incidence was additionally partitioned into the pre-ascertainment interval [0, 12) months, in which no recurrence can be detected by construction, and the ascertainment interval (≥ 12 months), in which the endpoint becomes observable (**Table S3b**). Because both observed recurrences occurred at the 12-month visit, all of the albendazole arm's events and event-bearing person-time fall in the ascertainment interval, where the combination arm contributed only 3.3 person-years (versus 24.1 in the albendazole arm) and no events. The absence of recurrence in the combination arm is therefore a direct consequence of its truncated post-12-month person-time rather than of a demonstrably lower hazard.

Table S3b. Recurrence incidence stratified by follow-up interval, by prophylaxis arm

Arm	Follow-up interval	Events	Person-years	Rate / 100 py	95% Poisson CI
Albendazole	[0, 12) months	0	29.5	0.00	[0.00, 12.50]
Albendazole	≥ 12 months	2	24.1	8.30	[1.01, 30.00]
Albendazole + Praziquantel	[0, 12) months	0	22.7	0.00	[0.00, 16.27]
Albendazole + Praziquantel	≥ 12 months	0	3.3	0.00	[0.00, 110.67]

Note: py = person-years. Person-time is partitioned within patient at the 12-month boundary: the pre-ascertainment contribution is min(follow-up, 12)/12 years and the ascertainment contribution is max(follow-up − 12, 0)/12 years. Rates are per 100 person-years with exact (gamma-based) Poisson 95% confidence intervals; a zero-event interval has a lower bound of zero. The whole-follow-up totals (albendazole 53.6 py, combination 26.0 py) reported in the main article are recovered by summing the two intervals within each arm. This table is produced by Part B of the analysis script (Supplementary File 2, `analysis.R`).

S4. Distribution of Follow-up Duration

Follow-up differed systematically between arms by virtue of the protocol's staggered observation window: the combination-treated patients were managed over a shorter window (median 10 months, range 6–18) than the monotherapy patients (median 24 months, range 6–24). Figure S2 makes this asymmetry directly visible and provides the structural

https://doi.org/10.3390/biomedicines14081734

context for the missingness pattern documented in Section S7 and for the interpretation of the 12-month landmark cavity-obliteration contrast.

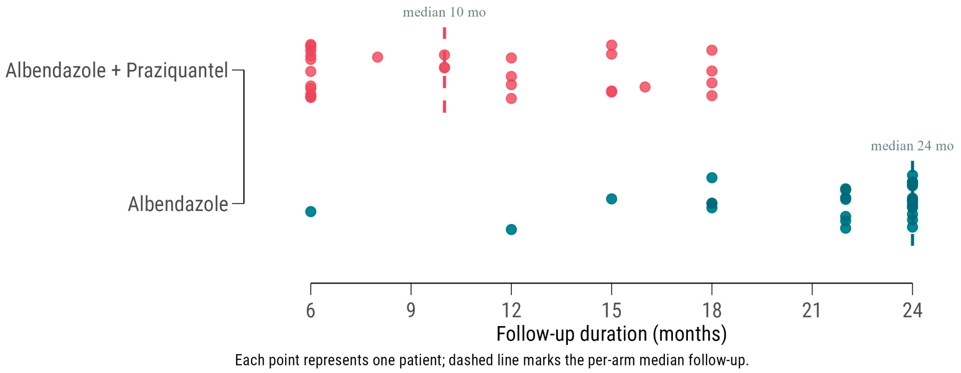

Figure S2. Distribution of follow-up duration (months) by prophylaxis arm. Dashed lines indicate the per-arm median follow-up duration. Histogram bins are three months wide.

To make the structural ascertainment pattern explicit in tabular form, the per-visit denominator was tabulated for each scheduled visit, with a patient defined as "reached" at visit V if the documented follow_up_months was greater than or equal to V (Table S4). The per-visit denominator trajectory is shown as a stepped line plot in the main article (Figure 6, fig0_followup_flow.png); the table below provides the exact denominators and per-arm percentages that condition every subsequent comparative analysis.

Table S4. Per-visit denominator by prophylaxis arm. Reached = documented follow_up_months ≥ scheduled visit.

Scheduled visit	Albendazole, *n* / 30 (%)	Albendazole + Praziquantel, *n* / 30 (%)	Total reached
0.5 mo	30 (100%)	30 (100%)	60
1 mo	30 (100%)	30 (100%)	60
2 mo	30 (100%)	30 (100%)	60
3 mo	30 (100%)	30 (100%)	60
6 mo	30 (100%)	30 (100%)	60
12 mo	29 (97%)	13 (43%)	42
18 mo	27 (90%)	4 (13%)	31
24 mo	16 (53%)	0 (0%)	16

S5. Propensity-score Overlap Diagnostic

A parsimonious logistic propensity score (PS) was estimated from age, number of cysts, baseline largest cyst size, rural residence and baseline AST. The covariate set was chosen for events-per-variable (EPV) efficiency rather than completeness: with 30 treated patients the five-covariate specification yields EPV ≈ 6, which is at the acceptable lower bound for a logistic PS. Adding the three per-organ cyst counts would drop EPV to ≈ 3.75, below the conventional 5–10 floor; the imbalance the per-organ counts expose (Table S1) is therefore addressed by an explicit caveat in the main article's Discussion rather than by an under-powered PS extension.

The propensity-score distributions by arm and the region of common support are shown in Figure S3. The two PS distributions overlap only over the interval [0.170, 0.889]; twelve of 60 patients (20%) fall outside this region. Limited overlap means residual confounding cannot be fully removed by any method, and all adjusted estimates remain observational rather than causal.

https://doi.org/10.3390/biomedicines14081734

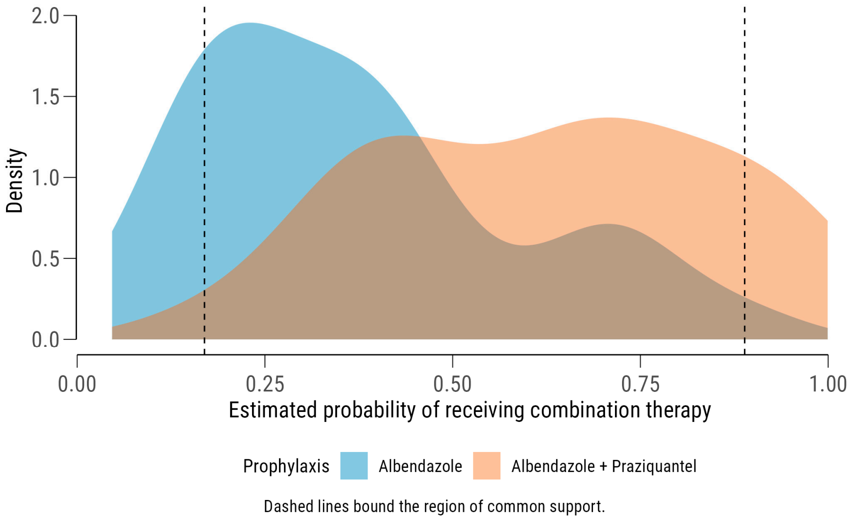

Figure S3. Estimated propensity-score densities by arm. Dashed lines bound the region of common support, defined by the intersection of the per-arm propensity-score ranges; 12 of 60 patients fall outside this region.

S6. Covariate Balance before and after Inverse-probability Weighting

To assess whether inverse-probability-of-treatment weighting (IPW; ATE weights) reduced the baseline imbalances, weighted standardized mean differences were computed for the five PS covariates and contrasted with the unweighted SMDs (Table S5; Figure S4). All five covariates moved closer to balance after weighting, with three of five (cyst burden, baseline largest cyst size, and rural residence) falling below the |SMD| = 0.10 threshold; baseline AST remained the most residually imbalanced covariate (weighted SMD = 0.207). The inverse-probability-weighted effective sample sizes were 19.1 in the albendazole arm and 23.6 in the combination arm (from 30 each), indicating substantial weight concentration. Apparent post-weighting balance under poor overlap can reflect a few dominant weights and must be read jointly with the effective sample size; both are reported here for transparency.

Table S5. Standardized mean differences before versus after IPW (ATE weights).

Covariate	Unadjusted SMD	IPW (ATE) SMD	Improved by IPW
Age	−0.415	−0.135	yes
Number of cysts	0.414	0.100	yes
Cyst size (baseline, mm)	0.381	0.062	yes
Village residence	0.474	−0.025	yes
Baseline AST (U/L)	0.478	0.207	yes

https://doi.org/10.3390/biomedicines14081734

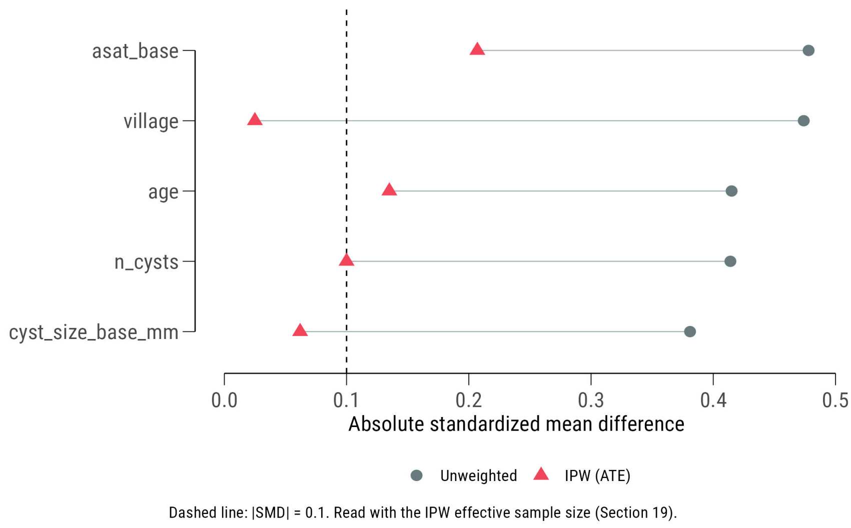

Figure S4. Absolute standardized mean differences before (grey circles) and after IPW (red triangles), with the |SMD| = 0.10 threshold shown as a dashed line. All five propensity-score covariates moved closer to balance under weighting.

S7. Structural versus Sporadic Missingness

Missing values arising from the differential, protocol-driven follow-up were treated as structural (missing not at random by construction) rather than as random attrition and were not imputed. Figure S5 displays the percentage of missing values across the four analyte families (haemoglobin, eosinophils, AST, ALT) by scheduled visit and arm. The diagonal staircase visible in the combination-arm panel after the 6-month visit reflects the protocol follow-up windows and is not a candidate for imputation; analyses were confined to visits each arm actually reached. Within reached visits, sporadic missingness was 7.1% in the albendazole arm and 20.0% in the combination arm across all four analyte families, reflecting the higher administrative attrition associated with the shorter combination-arm follow-up window.

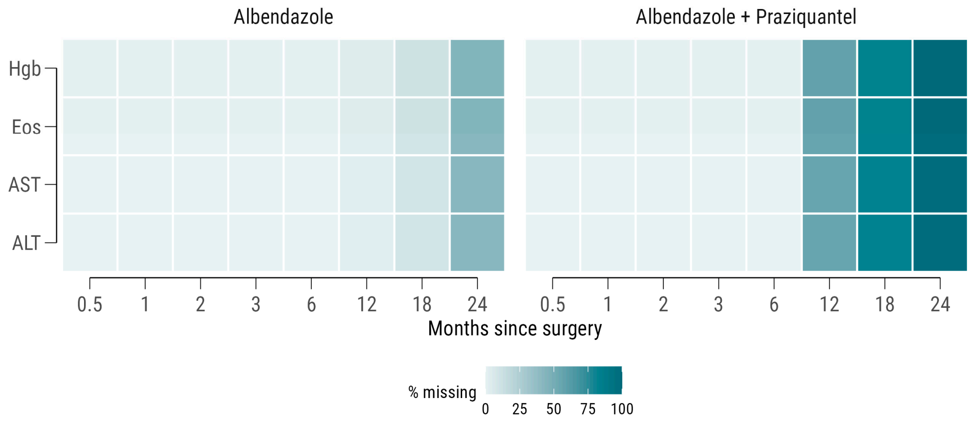

Figure S5. Missingness map by analyte family, scheduled visit and arm. High values at later visits in the combination arm are structural, driven by the protocol follow-up window, and should not be interpreted as random attrition.

S8. Directed Acyclic Graph for the Hepatotoxicity Adjustment Set

The adjustment set for the hepatotoxicity contrast was specified a priori through the directed acyclic graph shown in Figure S6. Under the encoded causal assumptions, the

https://doi.org/10.3390/biomedicines14081734

minimal sufficient adjustment set for the total effect of treatment on hepatotoxicity comprises baseline AST and age (the back-door paths Treatment ← Baseline AST → Hepatotoxicity and Treatment ← Age → Hepatotoxicity are both blocked by conditioning on these two covariates; the encoded Treatment-only effects of cyst burden and residence do not open additional back-door paths). The DAG-aligned model {treatment + baseline AST + age} is therefore the primary adjusted specification reported in the main article; the prior {treatment + baseline AST + baseline ALT} specification is retained as a sensitivity model (baseline ALT is not encoded as a confounder in the DAG but co-measures hepatocellular injury), and a kitchen-sink {treatment + baseline AST + baseline ALT + age} model is added for completeness. The three specifications agree directionally (OR 1.45, 1.40, and 1.48, respectively), confirming that the choice between them is robustness rather than confounding-control. The DAG encodes assumptions rather than data; it is scoped to the hepatotoxicity endpoint that is actually adjusted, and the recurrence endpoint is intentionally left unadjusted (two events). Differential follow-up acts through selection and censoring rather than as a classical confounder and is therefore represented separately, through the per-visit denominator tabulation (Section S4), the landmark analysis, and the tipping-point sensitivity in the main article.

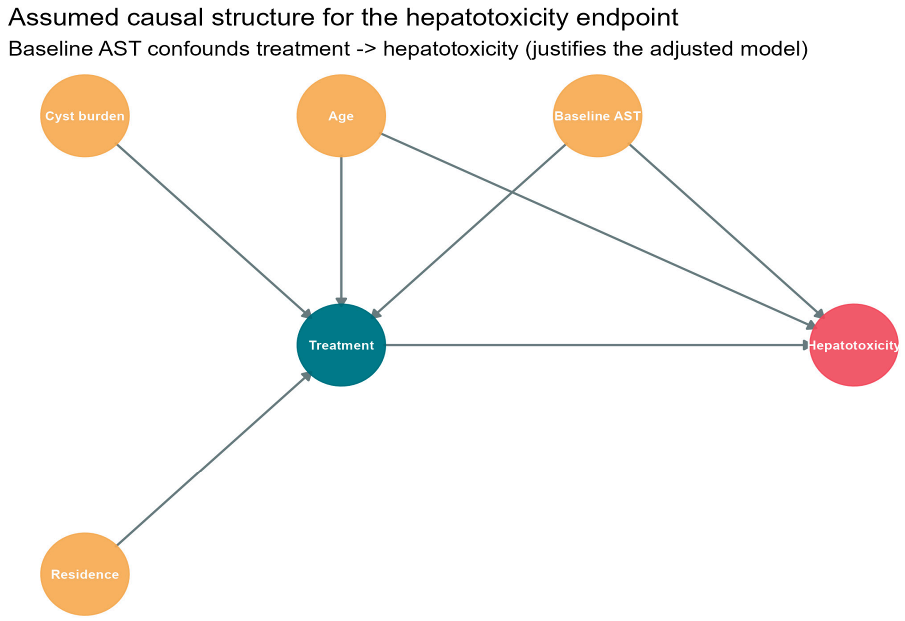

Figure S6. Assumed causal structure for the hepatotoxicity endpoint. The minimal sufficient adjustment set for the total effect of treatment on hepatotoxicity comprises baseline AST and age; conditioning on both blocks all back-door paths under the encoded structure.

S9. Extended Statistical Methods

This section provides the full technical specification of the analytic procedures summarised at the level of principle in the main article's Statistical Analysis section, in response to the reviewers' request that the advanced statistical detail be relocated here to improve the readability of the main text. The software environment (R 4.5.1) and the annotated code (Supplementary File 2) are described under Reproducibility below.

S9.1. Descriptive statistics and test selection

Continuous variables were summarised as mean ± standard deviation when both arms satisfied the Shapiro-Wilk normality criterion and otherwise as median with interquartile range. The between-arm test was selected accordingly: Student's or Welch's t-test

on the normal path (with the mean difference and its 95% confidence interval as the accompanying effect size) and the Mann-Whitney U test on the rank-based path (with the Hodges-Lehmann location shift and its 95% confidence interval). Categorical variables were compared with Pearson's chi-square test, replaced by Fisher's exact test whenever more than 20% of expected cell counts fell below five or any expected count fell below one. Given the non-randomized design, all between-arm p-values are interpreted as descriptive summaries of the observed distributions rather than as tests of a sampling null.

S9.2. Baseline balance

Because allocation was non-randomized, baseline covariate balance was quantified with standardized mean differences (SMDs), treating |SMD| > 0.10 as the design-appropriate imbalance flag irrespective of the accompanying significance test. The full fifteen-covariate balance table and Love plot are reported in Section S1.

S9.3. Recurrence endpoint: person-time, landmark, tipping-point, and interval-censored models

Recurrence-free survival was estimated by the Kaplan-Meier method and compared with the log-rank test, both reported for transparency only because the differential follow-up and the two-event count render them uninformative. The design-appropriate summaries were: (i) person-time incidence rates per 100 person-years with exact Poisson 95% confidence intervals, additionally partitioned at the 12-month ascertainment boundary (Section S3, Table S3b); (ii) a primary-window contrast restricted to the subcohort reaching the 12-month landmark, summarised as an absolute risk difference on the Newcombe hybrid-score interval; and (iii) a tipping-point sensitivity analysis over the patients with less than 12 months of follow-up (the unascertained set), recomputing the contrast under the worst-case assumption that all such patients had recurred. A semi-parametric interval-censored proportional-hazards model was fitted as a further sensitivity analysis; with zero events in the combination arm it separated and yielded no usable hazard ratio, which was itself treated as confirmation that frequentist time-to-event modelling of this endpoint is uninformative.

S9.4. Bayesian conjugate Beta-Binomial re-analysis

To obtain a finite, interpretable summary of the recurrence contrast under a zero-event arm, each arm's event probability was assigned an independent Beta prior and updated to its closed-form Beta posterior. The uniform Beta(1,1) prior served as the primary analysis and the Jeffreys Beta(0.5,0.5) prior as a prior-sensitivity check. The posterior distributions of the risk difference, risk ratio, and odds ratio (combination versus albendazole) were obtained by Monte Carlo sampling with 200,000 draws. The estimand of primary interest was the posterior probability of benefit, defined as the posterior probability that the combination arm carries the lower true event probability; it was interpreted against conventional thresholds (0.95 for moderate and 0.975 for strong evidence). The same Beta-Binomial machinery was applied to the any-adverse-event and elevated-transaminase endpoints as a cross-check on the frequentist safety analysis.

S9.5. Safety endpoints: propensity score, IPW, DAG-based adjustment, and E-value

Confounding of the adequately powered safety endpoints was addressed by propensity-score (PS) adjustment and inverse-probability-of-treatment weighting (IPW, average-treatment-effect weights). The parsimonious PS logistic model was estimated from age, number of cysts, baseline largest cyst size, rural residence, and baseline AST, a five-covariate specification chosen for events-per-variable efficiency (Section S5). Covariate overlap and the region of common support were assessed graphically (Section S5), and post-weighting balance was verified using weighted SMDs and the inverse-probability-weighted effective sample size (Section S6). The primary adjusted model for elevated

transaminases was defined a priori through a directed acyclic graph (Section S8): under the encoded assumptions the minimal sufficient adjustment set for the total effect of treatment on hepatotoxicity comprises baseline AST and age, giving the model {treatment + baseline AST + age}; a {baseline AST, baseline ALT} model and a {baseline AST, baseline ALT, age} model were retained as sensitivity specifications. Sensitivity to unmeasured confounding was gauged with the E-value of the adjusted odds ratio (VanderWeele and Ding closed form). Because the adjusted confidence interval for elevated transaminases already includes the null, the E-value is reported for the point estimate only, as a descriptive gauge of its fragility, and not for the confidence limit; when a confidence interval includes the null, no unmeasured confounding is required for compatibility with the null, so an E-value for the confidence limit does not constitute evidence of robustness.

S9.6. Within-arm transaminase dynamics

Within-arm transaminase elevation from baseline to peak on-treatment value was assessed with paired Wilcoxon signed-rank tests. Longitudinal transaminase burden over the active monitoring window (0-3 months) was quantified as the incremental area under the curve above baseline by the trapezoidal rule, compared across arms with Mann-Whitney U tests and baseline-adjusted linear regression. A linear mixed-effects model with a random patient intercept was fitted to AST over 0.5-3 months to evaluate the group-by-time interaction, adjusted for baseline levels.

S9.7. Cavity obliteration and missing data

Time to obliteration of the postoperative residual cavity was estimated by the Kaplan-Meier method. Because the all-time comparison is confounded by the arms' differential follow-up, interpretation was anchored a priori on the 6-month landmark, at which both arms were fully observed, with obliteration probabilities compared by a two-sample z-test using Greenwood standard errors; the 12-month landmark and the crude all-time comparison are reported for transparency only. Missing values arising from the differential, protocol-driven follow-up were treated as structural (missing not at random by construction) and were not imputed; analyses were confined to the visits each arm actually reached, and the structural-versus-sporadic missingness pattern is characterised in Section S7.

Reproducibility

Every table and figure in this supplement, and every numerical result in the main article, was produced by a single annotated R script (`analysis.R`, which includes the per-visit denominator block, the primary-window subcohort analysis, the DAG-aligned hepatotoxicity adjustment with its E-value, and the Bayesian posterior benchmark comparator) supplied as Supplementary File 2. The script is fully self-contained: it reads the cleaned wide-format dataset `data-echinococcosis.csv` (60 patients × 108 variables) directly, computes all derived analytic variables in place, and writes every figure to a `figures/` subfolder at 300 dpi on a white background. R software (version 4.5.1; R Foundation for Statistical Computing, Vienna, Austria) was used, with the tidyverse, survival, broom, scales and sessioninfo packages as core dependencies; the optional `lme4`, `parameters`, `naniar`, `icenReg`, `rstanarm`, `dagitty` and `ggdag` packages are loaded under guarded blocks and degrade gracefully when absent. Analytic decisions, including test-selection logic, balance thresholds, the rationale for restricting propensity-score adjustment to the safety endpoints, and the DAG-implied adjustment-set derivation, are documented in the script header.

The individual patient-level data underlying `data-echinococcosis.csv` are confidential and cannot be shared publicly, because a single-centre cohort of 60 patients with a rare

https://doi.org/10.3390/biomedicines14081734

disease cannot be robustly de-identified against re-identification (see the Data Availability Statement in the main article). To allow the code to be executed and verified without disclosing any patient record, a synthetic surrogate dataset of identical structure (`data-echinococcosis-SYNTHETIC.csv`), generated by random draws from the documented variable ranges and factor levels, is provided together with the data dictionary and the script. The synthetic dataset contains no real patient information and does not reproduce the study results; running the pipeline against it demonstrates that the code executes and returns outputs of the correct form, whereas the reported numbers are recovered only from the real data held by the authors.

https://doi.org/10.3390/biomedicines14081734
