# Supplementary material for: Postoperative Prophylaxis of Cystic Echinococcosis with Albendazole and Praziquantel: A Retrospective Cohort Study
Source: Biomedicines. 2026 Jul 31;14(8):1734. doi: 10.3390/biomedicines14081734 (PMC13510079; doi:10.3390/biomedicines14081734)
Supplement: Supplementary file 1 [file biomedicines-14-01734-s001.zip › Supplementary File S3.pdf]

biomedicines

MDPI

Supplementary File S3: STROBE Checklist for Cohort Studies

Postoperative Prophylaxis of Cystic Echinococcosis with Albendazole and Praziquantel: A Retrospective Observational Cohort Study

Galya Popova-Daskalova[1,6,*], Kostadin Kostadinov[2,3], Krasimira Eneva[1,6], Albena Masarlieva[1,6], Dimitar Vuchev[1], Angel Uchikov[4,6], Lyubka Aleksova[4,6], Nikolay Nikolov[4,6], Tanya Nedkova[5]

[1] Department of Infectious Diseases, Parasitology and Tropical Medicine, Section of Parasitology, Medical University of Plovdiv, Bulgaria, galiya.popova@mu-plovdiv.bg, krasimira.eneva@mu-plovdiv.bg, albena.masarlieva@mu-plovdiv.bg, dimitar.vuchev@mu-plovdiv.bg
[2] Department of Social Medicine and Public Health, Medical University of Plovdiv, 4002 Plovdiv, Bulgaria, kostadinr.kostadinov@mu-plovdiv.bg
[3] Environmental Health Division, Research Institute at Medical University of Plovdiv, 15-A "Vasil Aprilov" Blvd, 4002 Plovdiv, Bulgaria
[4] Department of Special Surgery, Medical University of Plovdiv, Bulgaria, angel.uchikov@mu-plovdiv.bg, lyubka.aleksova@mu-plovdiv.bg, nikolay.st. nikolov@mu-plovdiv.bg
[5] Faculty of Medicine, Medical University of Plovdiv, Bulgaria, tanyanedkova26@gmail.com
[6] "St. George" University Hospital, Plovdiv, Bulgaria
*Correspondence: galiya.popova@mu-plovdiv.bg

The STROBE statement (Strengthening the Reporting of Observational Studies in Epidemiology) provides a 22-item checklist for the transparent reporting of observational studies. The checklist below applies the cohort-study variant to the present manuscript, with cross-references to the manuscript section in which each item is addressed. Where an item is addressed jointly in the main article and in Supplementary File 1, both locations are given.

#	STROBE item	Recommendation	Location in manuscript
1(a)	Title and abstract	Indicate the study's design with a commonly used term in the title or the abstract	Title; Abstract — Methods
1(b)	Title and abstract	Provide in the abstract an informative and balanced summary of what was done and what was found	Abstract

https://doi.org/10.3390/biomedicines14081734

Introduction			
2	Background/rationale	Explain the scientific background and rationale for the investigation being reported	Introduction §1–§3
3	Objectives	State specific objectives, including any prespecified hypotheses	Introduction, final paragraph
Methods			
4	Study design	Present key elements of study design early in the paper	Methods — Study Design and Setting
5	Setting	Describe the setting, locations, and relevant dates, including periods of recruitment, exposure, follow-up, and data collection	Methods — Study Design and Setting; Methods — Follow-up and Outcome Ascertainment
6(a)	Participants	Cohort study — Give the eligibility criteria, and the sources and methods of selection of participants. Describe methods of follow-up	Methods — Participants and Eligibility Criteria; Methods — Follow-up and Outcome Ascertainment
6(b)	Participants	Cohort study — For matched studies, give matching criteria and number of exposed and unexposed	Not applicable (no matched design); allocation rationale described under Methods — Participants and Eligibility Criteria, with confounding-by-indication acknowledged
7	Variables	Clearly define all outcomes, exposures, predictors, potential confounders, and effect modifiers. Give diagnostic criteria, if applicable	Methods — Outcomes, Variables and Data Sources; Supplementary File 1, Section S8 (DAG for the hepatotoxicity adjustment set)
8*	Data sources/measurement	For each variable of interest, give sources of data and details of methods of assessment (measurement). Describe comparability of assessment methods if there is more than one group	Methods — Outcomes, Variables and Data Sources; Methods — Follow-up and Outcome Ascertainment
9	Bias	Describe any efforts to address potential sources of bias	Methods — Bias and Study Size; Supplementary File 1, Sections S1, S4, S5, S6
10	Study size	Explain how the study size was arrived at	Methods — Bias and Study Size (convenience cohort; no *a priori* sample-size calculation; underpowered for recurrence endpoint with 2 events)
11	Quantitative variables	Explain how quantitative variables were handled in the analyses. If applicable, describe which groupings were chosen and why	Methods — Outcomes, Variables and Data Sources; Methods — Statistical Analysis
12(a)	Statistical methods	Describe all statistical methods, including those used to control for confounding	Methods — Statistical Analysis

https://doi.org/10.3390/biomedicines14081734

12(b)	Statistical methods	Describe any methods used to examine subgroups and interactions	Methods — Statistical Analysis (LMM for group × time interaction in transaminases)
12(c)	Statistical methods	Explain how missing data were addressed	Methods — Statistical Analysis (structural missingness, no imputation); Supplementary File 1, Section S7
12(d)	Statistical methods	Cohort study — If applicable, explain how loss to follow-up was addressed	Methods — Statistical Analysis (per-visit denominator tabulation; primary-window subcohort; tipping-point sensitivity); Supplementary File 1, Section S4
12(e)	Statistical methods	Describe any sensitivity analyses	Methods — Statistical Analysis (tipping-point analysis; interval-censored PH sensitivity; Bayesian re-analysis under uniform and Jeffreys priors; E-value for adjusted safety OR; three alternative hepatotoxicity adjustment specifications)
Results			
13(a)	Participants	Report numbers of individuals at each stage of the study — for example, numbers potentially eligible, examined for eligibility, confirmed eligible, included in the study, completing follow-up, and analysed	Results — Patient Characteristics (Table 1); Methods — Follow-up; Main article Figure 1 (per-visit denominators); Supplementary File 1, Table S4 (per-visit denominator table)
13(b)	Participants	Give reasons for non-participation at each stage	Methods — Follow-up and Outcome Ascertainment (structural attrition driven by the staggered protocol observation window)
13(c)	Participants	Consider use of a flow diagram	Main article Figure 1 (per-visit denominator trajectory by arm); Supplementary File 1, Section S4
14(a)	Descriptive data	Give characteristics of study participants (e.g., demographic, clinical, social) and information on exposures and potential confounders	Results — Patient Characteristics (Table 1); Supplementary File 1, Table S1 (full baseline balance, 15 covariates)
14(b)	Descriptive data	Indicate number of participants with missing data for each variable of interest	Supplementary File 1, Section S7
14(c)	Descriptive data	Cohort study — Summarise follow-up time (e.g., average and total amount)	Methods — Follow-up; Supplementary File 1, Section S4 (median 24 vs 10 months; full per-visit denominator)
15*	Outcome data	Cohort study — Report numbers of outcome events or summary measures over time	Results — Disease Recurrence; Results — Safety and Tolerability (Table 5)
16(a)	Main results	Give unadjusted estimates and, if applicable, confounder-adjusted estimates and their precision (e.g., 95%	Results — Disease Recurrence (Newcombe RD, Katz RR, person-time IR with Poisson 95% CI, primary-window

https://doi.org/10.3390/biomedicines14081734

		confidence interval). Make clear which confounders were adjusted for and why they were included	subcohort RD); Results — Safety (DAG-aligned OR with 95% CI and three sensitivity specifications); Supplementary File 1, Sections S2, S5, S6
16(b)	Main results	Report category boundaries when continuous variables were categorised	Results — Cyst Burden (largest-cyst dichotomy at 100 mm; size band at 100 mm); Methods — Outcomes (hepatotoxicity severity grades at 2× and 3× ULN)
16(c)	Main results	If relevant, consider translating estimates of relative risk into absolute risk for a meaningful time period	Results — Disease Recurrence (risk differences; 1 − KM at 12, 18, 24 months)
17	Other analyses	Report other analyses done — e.g., analyses of subgroups and interactions, and sensitivity analyses	Results — Disease Recurrence (tipping-point sensitivity; Bayesian re-analysis under both priors with posterior probability and credible interval, plus the conventional 0.95 benchmark; primary-window subcohort); Results — Safety (E-value for the DAG-aligned OR; alternative hepatotoxicity adjustment specifications)
Discussion			
18	Key results	Summarise key results with reference to study objectives	Discussion, opening paragraphs and Conclusions
19	Limitations	Discuss limitations of the study, taking into account sources of potential bias or imprecision. Discuss both direction and magnitude of any potential bias	Strengths and Limitations (non-randomization, baseline imbalance, structural differential follow-up, sparse-events separation, lower historical praziquantel dose, single-centre, short follow-up relative to natural history of CE)
20	Interpretation	Give a cautious overall interpretation of results considering objectives, limitations, multiplicity of analyses, results from similar studies, and other relevant evidence	Discussion (full); Conclusions (explicitly non-causal interpretation)
21	Generalisability	Discuss the generalisability (external validity) of the study results	Strengths and Limitations (single-centre convenience cohort; historical praziquantel dose; need for prospective evaluation at current WHO-recommended dose)
Other Information			
22	Funding	Give the source of funding and the role of the funders for the present study and, if applicable, for the original study on which the present article is based	Funding

https://doi.org/10.3390/biomedicines14081734
